# Early biological and psychosocial factors associated with PTSD onset and persistence in youth

**DOI:** 10.1080/20008066.2024.2432160

**Published:** 2024-12-09

**Authors:** Kimberley C. Williams, Nto J. Nto, Esmé Jansen van Vuren, Farhanah N. Sallie, Keneilwe Molebatsi, Kayla S. Kroneberg, Aqeedah A. Roomaney, Muneeb Salie, Jacqueline S. Womersley

**Affiliations:** a Department of Psychiatry and Mental Health, Faculty of Health Sciences, University of Cape Town, Cape Town, South Africa; b Neuroscience Institute, University of Cape Town, Cape Town, South Africa; c Department of Psychiatry, Faculty of Medicine and Health Sciences, Stellenbosch University, Cape Town, South Africa; d South African Medical Research Council/Stellenbosch University Genomics of Brain Disorders Extramural Unit, Faculty of Medicine and Health Sciences, Stellenbosch University, Cape Town, South Africa; e Department of Anatomy, Faculty of Basic Medical Sciences, University of Nigeria, Nsukka, Nigeria; f Hypertension in Africa Research Team (HART), North-West University, Potchefstroom, South Africa; g SAMRC Extramural Unit for Hypertension and Cardiovascular Disease, Faculty of Health Sciences, North-West University, Potchefstroom, South Africa; h Wits Integrated Molecular Physiology Research Initiative, Wits Health Consortium (PTY) Ltd, School of Physiology, Faculty of Health Sciences, University of The Witwatersrand, Johannesburg, South Africa; i School of Physiology, Faculty of Health Sciences, University of The Witwatersrand, Johannesburg, South Africa; j Department of Psychiatry, Faculty of Medicine, University of Botswana, Gaborone, Botswana; k Western Cape Department of Health, False Bay District Hospital, Cape Town, South Africa

**Keywords:** PTSD, children, adolescents, biological factors, psychological factors, social factors, TEPT, niños, adolescentes, factores biológicos, factores psicológicos, factores sociales

## Abstract

**Background:** Posttraumatic stress disorder (PTSD) is a debilitating mental health condition that can develop after experiencing or witnessing a traumatic event. While considerable research has investigated PTSD in adults, little is known about the biological, psychological, and social factors that contribute to its onset, development, and persistence in youth.

**Methods:** This systematic review followed the Preferred Reporting Items for Systematic Reviews and Meta-Analyses (PRISMA) guidelines to identify longitudinal studies examining factors associated with PTSD status and symptom severity in children and adolescents. Literature searches were conducted in PubMed, Scopus, and Web of Science, yielding 24 eligible studies after screening.

**Results:** The included studies identified various biological factors associated with paediatric PTSD, including dysregulation of the hypothalamic–pituitary–adrenal axis, brain structural alterations, and physiological markers such as heart rate. Psychological factors, including depression, trauma appraisals, coping styles, and cognitive deficits predicted PTSD symptom development. Social factors included parental PTSD, family environment, and cultural influences. Many studies highlighted the importance of the interplay between these biological, psychological, and social factors in the manifestation of PTSD in youth.

**Conclusion:** This review synthesises evidence that PTSD development in youth is influenced by a complex array of neurobiological vulnerabilities, psychological processes, and environmental factors. Longitudinal, multi-dimensional studies are needed to further elucidate personalised risk profiles and trajectories, which can inform targeted prevention and intervention strategies for PTSD in youth.

## HIGHLIGHTS

- An integrative biopsychosocial approach to understanding PTSD risk in children and adolescents is needed. Stress response dysregulation, autonomic nervous system activity, and brain structure and function were the most widely studied biological factors but showed inconsistent findings that may reflect differences in demographic factors, study design and the nature and duration of the trauma exposure.
- Psychosocial factors were less well studied but identified trauma appraisals, cognitive function profiles and coping styles as individual-level risk factors. Parental psychopathology was associated with higher PTSD risk.
- Differences in the range of factors investigated in studies of youth and adults highlight the need for research focussed on younger populations. Future studies should employ multidimensional approaches, include diverse populations, have increased representation of low- and middle-income countries, and consider risk factors according to different developmental stages.

## GRAPHICAL ABSTRACT

**Figure:**
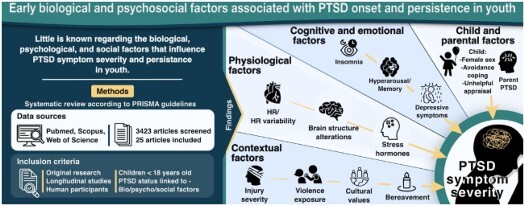

The systematic review of 24 articles identified a range of biopsychosocial factors that may influence the onset and persistence of PTSD in children and adolescence. Multiple gaps in the literature were also identified and should be addressed by well-powered multimodal studies that carefully consider developmental stage and include participants that more closely reflect the diverse populations affected by trauma globally.

## Abstract (es)

**Antecedentes:** El trastorno de estrés postraumático (TEPT) es una condición mental debilitante que puede desarrollarse tras experimentar o presenciar un acontecimiento traumático. Aunque se han realizado numerosas investigaciones sobre el TEPT en adultos, se sabe poco sobre los factores biológicos, psicológicos y sociales que contribuyen a su aparición, desarrollo y persistencia en los jóvenes.

**Métodos:** Esta revisión sistemática siguió las directrices de los Elementos de Reporte Preferidos para Revisiones Sistemáticas y Metaanálisis (PRISMA por sus siglas en inglés) para identificar estudios longitudinales que examinaran los factores asociados con el estado del TEPT y la gravedad de los síntomas en niños y adolescentes. Se realizaron búsquedas bibliográficas en PubMed, Scopus y Web of Science, y tras el cribado se obtuvieron 24 estudios elegibles.

**Resultados:** Los estudios incluidos identificaron diversos factores biológicos asociados con el TEPT pediátrico, incluida la desregulación del eje hipotálamo-hipófisis-suprarrenal, alteraciones estructurales cerebrales y marcadores fisiológicos como la frecuencia cardiaca. Los factores psicológicos, como la depresión, las evaluaciones del trauma, los estilos de afrontamiento y los déficits cognitivos predijeron el desarrollo de síntomas de TEPT. Los factores sociales incluían el TEPT de los padres, el entorno familiar y las influencias culturales. Muchos estudios destacaron la importancia de la interacción entre estos factores biológicos, psicológicos y sociales en la manifestación del TEPT en los jóvenes.

**Conclusiones:** Esta revisión sintetiza la evidencia de que el desarrollo del TEPT en los jóvenes está influido por un complejo conjunto de vulnerabilidades neurobiológicas, procesos psicológicos y factores ambientales. Se necesitan estudios longitudinales y multidimensionales para dilucidar mejor los perfiles y trayectorias de riesgo personalizados, que pueden servir de base para estrategias de prevención e intervención específicas para el TEPT en jóvenes.

## 1. Background

Post-traumatic stress disorder (PTSD) is a psychological condition resulting from exposure to traumatic events such as combat, disasters, accidents, or violence (Mansour et al., 2023). Symptoms of PTSD include intrusive memories, heightened arousal, mood changes, and avoidance behaviours associated with the traumatic event (American Psychiatric Association, 2013). An estimated 43% of people experience trauma in their lifetime (Knipscheer et al., 2020), with a PTSD prevalence in the general population of approximately 3.9% (Koenen et al., 2017). Around one billion children between the ages of 2- and 17-years old experience abuse or trauma yearly (Copeland et al., 2007; Hillis et al., 2016; Lewis et al., 2019), with meta-analyses suggesting that 15.9% of children and adolescents, and 21.5% of preschoolers exposed to trauma, may develop PTSD, underscoring youth vulnerability to the disorder (Alisic et al., 2014; Woolgar et al., 2022).

Studies have explored why some individuals develop PTSD after a traumatic event while others do not. Research has identified several risk factors, including but not limited to, prolonged exposure to intense trauma, adverse childhood experiences, and psychological comorbidity (Aliev et al., 2020; Knipscheer et al., 2020). Neurobiological mechanisms, such as hypothalamic–pituitary–adrenal (HPA) axis dysregulation; changes in neurotransmitter systems; immunity; genetic predisposition; and structural changes in brain regions involved in fear processing and memory could also account for variation in trauma responses (Aliev et al., 2020; Hu et al., 2020; Magwai & Xulu, 2022).

Differences in the biopsychosocial pathways underlying PTSD risk may vary between children and adults. Childhood and adolescence involve significant neurobiological and psychosocial changes, creating vulnerability to adverse outcomes. Ongoing brain maturation, especially in the prefrontal cortex, increases sensitivity to stress in children and adolescents (Agorastos et al., 2019). Consequently, biological markers of PTSD in children and adolescents could represent the impact that trauma has on the developing brain. In comparison, the effects of trauma in adults involve established neurobiological pathways. In the psychosocial model, perceptions of the trauma, which are shaped by the environment, e.g. lack of social support, peer influences, feelings of helplessness, and fear of social rejection, may play a significant role in PTSD development among adolescents. Similar psychosocial factors may be at work in adults but occur in the context of a longer history of previous experiences and traumas (Cisler & Herringa, 2021).

While significant strides have been made in understanding PTSD, the disorder's complexities continue to pose substantial challenges as conflicting evidence and gaps persist (Yehuda et al., 2015). Despite extensive research, a consensus on specific molecular and genetic markers for predicting and diagnosing PTSD in children remains elusive (Agorastos et al., 2019). Environmental influences add another layer of uncertainty. The role of parental PTSD and parenting practices in the intergenerational transmission of trauma remains unclear (Morris et al., 2012). Additionally, the long-term mental health outcomes from single or cumulative trauma exposures during development are not fully understood (Agorastos et al., 2019; Cloitre et al., 2009). Notably, societal, familial, and cultural contexts significantly shape the perception, experience, and expression of PTSD. These contexts influence emotional expressiveness, coping strategies, and help-seeking behaviours, ultimately affecting symptom severity and offering therapeutic targets for intervention (Rousseau & Measham, 2007).

Many PTSD studies in children are cross-sectional, limiting causal inferences between PTSD and biopsychosocial variables (O’Donnell et al., 2014). Additionally, studies often overlook ethnic, cultural, socioeconomic, and gender diversity, reducing the generalisability and understanding of PTSD (McClendon et al., 2019). Most studies use quantitative data, but qualitative measures could provide deeper insights into children’s lived experiences (Etherington, 2007). Studies often aggregate different trauma types, potentially distorting specific trauma effects. Tailored interventions and efficacy assessments across diverse settings are lacking, and long-term tracking of participants is rare. These gaps highlight the need for comprehensive, inclusive research to better understand childhood PTSD.

Several reviews in recent years have provided valuable insights into risk factors that impact child and adolescent PTSD. However, they have been relatively narrow in focus. For example, published reviews examined psychological (Memarzia et al., 2021), epidemiological (Yu et al., 2020), parenting behaviours (Williamson et al., 2017) and trauma appraisals (Mitchell et al., 2017) in isolation. Other reviews considered a broader range of factors but in response to specific traumas such as hospitalisation (de Pellegars et al., 2024; Triantafyllou & Matziou, 2019) or disasters and pandemics (Newnham et al., 2022). Furthermore, these reviews included cross-sectional studies and did not specifically limit their search to longitudinal studies. This systematic review summarises biological, psychological, and social factors associated with PTSD status and symptom severity as described by longitudinal studies involving youth from four to 18 years of age. This will provide novel insights by (a) focussing on a broader range of traumas, (b) considering biological, psychological and social factors and (c) examining whether these factors are associated with the development or course of PTSD. We use these insights to highlight gaps and inconsistencies in the literature, as well as important considerations for practice, policy, and research.

## 2. Methods

This systematic review used the Preferred Reporting Items for Systematic Reviews and Meta-Analyses (PRISMA) guidelines (Page et al., 2021).

### 2.1. Search strategy

The literature search of three databases, Pubmed, Scopus and Web of Science, was performed independently by two reviewers, JSW and MS, on 25 October 2023. The search string was designed to identify research publications reporting on the biological, psychological and social factors associated with PTSD onset or development in children and adolescents and comprised the following terms: ((biolog*) OR (neurobiolog*) OR (genom*) OR (DNA) OR (‘stress hormone’) OR (inflammat*)) AND ((psychosocial) OR (psycholog*) OR (social) OR (‘social support’) OR (relation*) OR (attachment) OR (family) OR (peer) OR (interpersonal) OR (integration) OR (‘social network’)) AND ((‘post-traumatic stress’) OR (‘posttraumatic stress’) OR (PTSD)) AND ((child*) OR (youth) OR (adolescen*) OR (teenage*) OR (girl) OR (boy)). The literature search was repeated on 2 May 2024 to identify newly published articles.

### 2.2. Eligibility criteria and selection process

**Figure 1.**
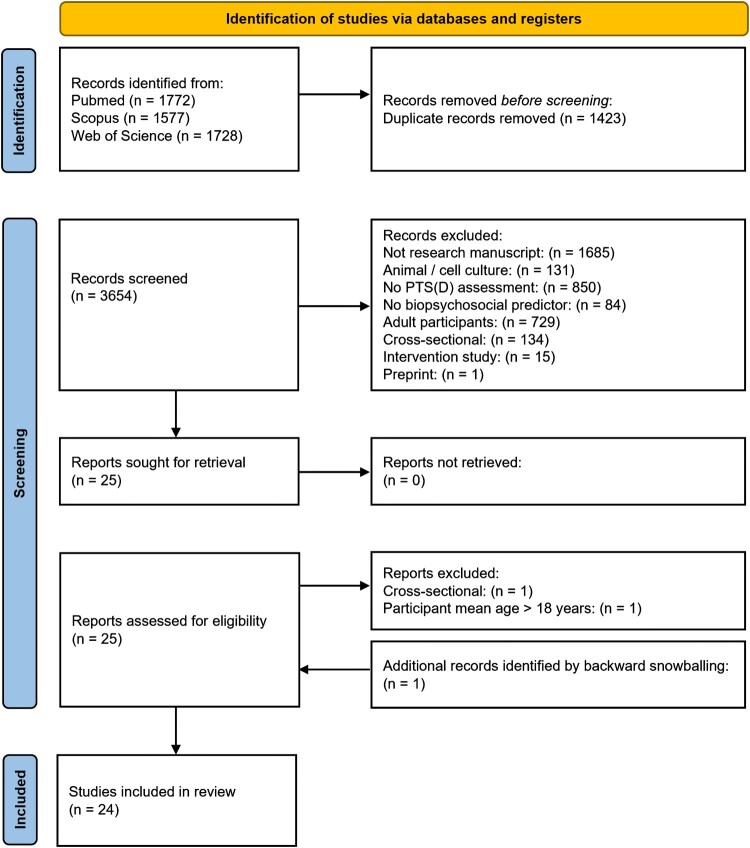
PRISMA flow diagram used to graphically summarise the number of studies identified, screened, eligible and included and excluded at each stage.

Search outputs from Pubmed (*n* = 1772), Scopus (*n* = 1577), and Web of Science (*n* = 1728) were uploaded to Rayyan, an online open-source software (Ouzzani et al., 2016). Rayyan was used to collate the database, scan for duplicates, and conduct preliminary abstract screenings. Potential duplicates (*n* = 1423) were manually checked and removed. To be included, studies had to report original research on longitudinal studies with human participants with an average age under 18 years at all timepoints and assess PTSD status or symptom severity related to biological, psychological, or social factors. Due to the polygenic nature of PTSD, where multiple individual variants make small contributions to overall risk, studies exclusively reporting candidate gene findings were excluded, as they are unlikely to provide fine discriminative prediction (Misganaw et al., 2019; Nievergelt et al., 2019). Abstracts were screened by at least two of three reviewers (JSW, AAR, and MS), with discrepancies resolved by consensus. The final selection was made after full-text reviews, including backward snowballing, resulting in 24 included articles (Figure 1).

### 2.3. Data extraction

Relevant study characteristics, aims and outcomes were extracted and summarised in Table 1. The data include the study design and setting (design, location of the study and/or site of participant recruitment, and the name of the parent study or cohort if applicable); sample characteristics (sample size, and sex, age, and ancestry of participants classified according to experimental group or study cohorts if applicable); aims; PTSD assessment measures; biopsychosocial factors, and the primary findings directly relevant to the systematic review topic. Findings beyond the scope of the review topic were excluded.

**Table 1.** Data extracted from studies examining biopsychosocial factors associated with PTSD in childhood and adolescence.

Study	Design and setting	Sample characteristics				Aims	Clinical PTSD / PTS measures	Biopsychosocial measures	Primary findings
		Sample size	Age* in years Mean (SD)	Sex (female)	Reported ancestry/ethnicity				
Delahanty et al. (2005)	Longitudinal study of children admitted to a trauma centre at Akron Children’s Hospital in the USA	*n* = 58	13.04 (3.05) Range: 8–18	31%	White (46%), African American (11%), mixed ancestry (1%)	To examine the relationship between urinary catecholamines and cortisol levels in children immediately following exposure to a first-time, acute duration traumatic event and development of PTSD symptoms at 6 weeks	6 weeks PTSD symptoms (CAPS-CA)	Baseline Urinary catecholamines and cortisol assessed within 12 hours of hospitalisation	Elevated levels of urinary cortisol and epinephrine within 12 hours of hospitalisation were positively associated with PTSD symptoms at 6 weeks post trauma.
Geng et al. (2019)	Longitudinal multiwave cohort study of adolescent earthquake survivors in China recruited to the Wenchuan Earthquake Adolescent Health Cohort (WEAHC) study	12 months *n* = 1407 18 months *n* = 1335 24 months *n* = 1361	15.01 (1.26)	55.4%	Chinese adolescents	To investigate the bidirectional associations between symptoms of PTSD, depression and insomnia assessed at 12-, 18- and 24-months post-earthquake exposure	12, 18 and 24 months PTSD symptoms (PTSD-SS)	Baseline Demographic characteristics (gender, grade in school, number of children in the family, residence) and degree of earthquake exposure (death, disappearance, and/or injury of family members, house or property loss or damage, and witnessing or hearing of tragic scenes) 12, 18 and 24 months Depressive (DSRS) and insomnia symptoms (two items extracted from the PSQI)	Insomnia predicted PTSD symptom severity in cross-sectional and longitudinal analyses. Depressive symptoms mediated the longitudinal insomnia-PTSD association. Depressive symptoms predicted longitudinal PTSD symptom severity.
Guo et al. (2017)	Longitudinal study in Australia of children admitted to the Royal Children’s Hospital in Melbourne and Mater Children’s hospital in Brisbane following TBI	*n* = 166	10.80 (2.55) Range: 6–14	27.7%	Not stated	To examine neurocognitive (sustained attention, selective attention, verbal learning, working memory) factors underlying the development of future PTSD symptoms following paediatric TBI	6 months PTSD symptoms (CAPS-CA)	2 months family socioeconomic status, and child medical and developmental history, academic performance, and social development. TBI-related measures including neuroimaging and GCS 3 months Cognitive measures including full scale IQ (WASI), selective and sustained attention (TEA-Ch), working memory (WISC-IV DS-b), verbal learning (CMS) and processing speed (CNT)	Younger age, female sex, and injury severity positively predicted 6-month total PTSD and avoidance symptoms. Poorer 3-month sustained attention predicted total 6-month PTSD and hyperarousal symptoms. Younger age and injury severity predicted re-experiencing and hyperarousal symptoms, respectively. Verbal learning in severe TBI and working memory in mild and moderate TBI were positively associated with PTSD symptom severity. Lower sustained attention and female sex in mild TBI, and lower IQ and younger age in moderate TBI were associated with PTSD symptom severity.
Haag et al. (2019)	Longitudinal study of trauma-exposed children and their parents across four hospital emergency departments in the United Kingdom.	Total *n* = 76 Baseline *n* = 70 Child trauma narrative *n* = 74 Joint trauma narrative *n* = 63	10.05 (0.22) Range: 6–13	39.5%	Caucasian (89.5%)	To examine whether mean heart rate (HR) and heart rate variability (HRV) at 1 month post trauma are associated with PTS symptom severity at 1, 3 and 6 months	1, 3 and 6 months PTSD symptoms (PTSD-RI Child Report)	Age, sex, and triage status at hospitalisation. 1 month: HR and HRV, defined as high (HFBP: 0.15–4 Hz) or low (LFBP 0.04–0.15) frequency bands, assessed at rest and during recount of trauma.	Higher 1-month mean HR and lower HFBP and LFBP HRV indices during child and parent-child joint trauma narratives were positively correlated with PTS severity at 1 and 3 months. Lower child and joint narrative HFBP, as well as joint narrative LFBP, were positively correlated with 6-month PTS scores. Only the 1-month mean HR during joint narrative and 3-month PTS relationship survived when accounting for 1-month PTS scores.
Heyn and Herringa (2019)	Longitudinal study of youth and adolescents in the USA recruited to the Youth PTSD Study of cortical markers of pediatric PTSD persistence and remission	Baseline Total *n* = 55 PTSD cases *n* = 28 of which remitter *n* = 10 non-remitter *n* = 18 Controls *n* = 27	Control 14.16 (2.70) PTSD Remitter 13.28 (3.45) PTSD non-remitter 14.21 (2.46) Range 8–18 years	Control 77.78% PTSD Remitter 50% PTSD Nonremitter 66.67%	Non-Hispanic White (63.6%), Hispanic or Latino (9.1%), African American (9.1%), unknown (16.4%)	To investigate relationships between trauma-related psychopathology and cortical development by examining longitudinal regional cortical thicknesses and surface areas (CSA) in relation to PTSD status and symptom trajectory	Baseline and 1-year: PTSD diagnosis (KSADS and CAPS-CA). Participants with PTSD completed the PTSD-RI.	Baseline only: Sex, index trauma variables, and parental income Baseline and 1-year: Age, Tanner developmental stage, psychopathology (KSADS, MFQ, SCARED) and trauma measures (CTQ, SLES) and IQ (WASI-II). MRI assessment of brain cortical surface area and thickness.	PTSD remission was associated with lower trauma exposure and baseline PTSD and depression symptom severity. PTSD status over time was associated with reduced left posterior cingulate CSA compared to non-exposed controls. PTSD persistence was associated with a reduced CSA over time across 5 brain regions: left ventrolateral prefrontal cortex, supramarginal gyrus and occipital pole and right superior parietal gyrus and precentral gyrus. PTSD remission was associated with increased left frontal pole cortical surface area and right ventromedial prefrontal cortex cortical thickness.
Heyn et al. (2022)	Longitudinal study of youth and adolescents in the USA recruited to the Youth PTSD Study of neuroimaging markers of PTSD	Total *n* = 97 PTSD cases *n* = 45 Controls *n* = 52	14.06 (2.91) Range: 7–17	Controls 59.61% PTSD 62.22%	Not reported	To investigate whether PTSD-associated differences in brain structure vary by sex and whether these differences predict symptom trajectory	Baseline PTSD diagnosis (KSADS and CAPS-CA) Baseline and one-year PTSD symptom severity in participants with PTSD (PTSD-RI)	Baseline Age, sex, Tanner developmental stage, index trauma variables, parental highest level of education, psychopathology (KSADS, MFQ, SCARED), trauma measures (CTQ, SLES) and IQ (WASI-II). MRI assessment of brain CSA and thickness and grey matter volume. Participants with PTSD provided information on psychotropic medication and psychotherapy history, and past or current comorbidities.	Compared to males, females with PTSD reported more hyperarousal symptoms and child abuse experience, right orbital gyrus grey matter volume, right frontomarginal gyrus and ventrolateral prefrontal cortex surface area. Females with PTSD had higher frontal pole cortical surface area compared to typically developing females. Baseline right orbital gyrus grey matter volume positively predicted 1-year PTSD symptom severity in females with the opposite effect seen in males.
Kolaitis et al. (2011)	Prospective study of the development and persistence of PTSD in youth hospitalised in Greece immediately following a road traffic accident	Baseline *n* = 57 1-month *n* = 57 6-month *n* = 48	10.9 (2.5) Range: 7–18	31.6%	Not reported	To explore predictors, including parental psychopathology and initial neuroendocrine responses, of the development and persistence of PTSD in children and adolescents immediately following a road traffic accident	1- and 6-months: PTSD diagnosis (PTSD Module of the K-SADS-PL)	Participant age, sex, GCS, and injury severity data collected. Maternal and paternal past and current psychopathology and PTSD symptom severity assessed using the SCL-90-R. Cortisol levels were measured in saliva collected within 24 hrs of the road traffic accident	Injury severity and higher 1-month maternal PTSD symptomatology predicted child PTSD at 3 months. Six-month PTSD diagnosis was positively associated with paternal past psychopathology and current paternal psychopathology and PTSD symptomology. Cortisol in saliva samples collected at 21h00 was higher in participants with PTSD at 6 months.
Linde-Krieger et al. (2024)	Prospective study of 225 caregiver-youth dyads recruited to an ongoing study of development and followed-up through the COVID-19 lockdown in the USA	*n* = 225	RSA assessment 6.11 (0.21) Caregiving assessment 12.24 (0.35) Pre-COVID PTS assessment 14.24 (0.50) COVID PTS assessment 15.23 (0.57)	49.8%	Latine (46.2%), Black (17.8%), White (11.6%), multiracial (24.4%)	To determine whether the interaction of childhood parasympathetic regulation and early adolescent caregiving environment influences PTS in adolescents during the COVID-19 pandemic, and to determine whether diathesis-stress or biological sensitivity to context models best explain these effects	Adolescence PTS symptom severity (YSR) at 14 and 15 years of age	Childhood RSA at 6 years Early adolescence Participants reported on caregiver attachment security (BSQ) and caregivers reported on internalising psychopathology (BSI)	Consistent with a biological sensitivity to context model, participants with higher childhood RSA were more sensitive to the valence of the caregiving environment, with higher attachment and lower caregiver internalising symptoms associated with lower PTS scores and *vice versa*.
Luo et al. (2012)	Study of female adolescents with and without exposure to the Wenchuan Earthquake in China	Total *n* = 84 Trauma-exposed with PTSD *n* = 32 Trauma-exposed without PTSD *n* = 32 Non-trauma-exposed controls *n* = 20	Trauma exposed with PTSD 13.81 (0.93) Trauma exposed without PTSD 13.84 (0.95) Non-trauma-exposed controls 14.40 (1.67) Range 12–15	100%	Not reported	To evaluate hair cortisol concentration (HCC) as a biomarker for altered hypothalamic-pituitary-adrenal axis activity in female adolescents with PTSD 7 months after the earthquake	7 months post-earthquake PTSD symptomology (Chinese version of the CRIES). Participants with CRIES scores >35 and <26 underwent PTSD diagnostic testing (SCID-IV for Axis I Disorders Patient and Non-Patient Editions, respectively)	7 months post-earthquake Cortisol concentration measurements in hair samples reflecting one pre, one peri and two post trauma exposure 3-month periods. Trauma-exposed participants reported on experience of certain traumatic events due to the earthquake	HCC did not differ between groups before the earthquake. Trauma exposure increased HCC at the time of the earthquake and was lower in trauma-exposed participants with vs. without PTSD at 2-4- and 5-7-months post-earthquake.
Marsac et al. (2017)	Prospective study of children recruited from a level 1 paediatric trauma centre in the USA and who were hospitalised for treatment of a potentially traumatic injury	Baseline *n* = 96 6 weeks *n* = 86 12 weeks *n* = 75	10.6 (1.7) Range: 8–13	38%	Black/African American (40.6%), White (52.1%), other (7.3%).	To examine whether biological, psychological, and environmental factors in the pre-, peri-, and post-trauma period predict PTS over time	Baseline, 6 and 12 weeks PTS symptom severity (CPSS)	Baseline HR, injury severity (GCS and ISS), trauma history (UCLA PTSD Index), trauma-specific appraisals of the injury events (ASC-Kids) and global negative posttraumatic appraisal (CPTCI) 6 and 12 weeks Coping (HICUPS)	Global and trauma-specific appraisals were associated with PTS at baseline but not 12-weeks. Avoidance and overall coping mediated the positive association between baseline and 12-week PTS symptom severity. Baseline HR was not associated with PTS scores at any time point.
McLaughlin et al. (2014)	Prospective study of adolescents exposed to media coverage and the shelter-in-place order following the 2013 Boston Marathon terrorist attack in the USA	*n* = 15	At fMRI scan 16.5 Range: 14.1–19.1 At PTS assessment 17.3 Range: 14.8–19.9	66.7%	White (46.7%), Black (26.7%), Latino (13.3%), other/biracial (13.3%)	To examine whether brain region activity during an fMRI task assessing emotional responding to aversive images predicts PTS symptom severity following indirect exposure to a terrorist attack	Pre-trauma exposure PTSD symptoms (YSR) Post-trauma exposure Boston Marathon Attack-associated PTS symptoms (IES-R)	Pre trauma exposure Internalising (CDI, MASC) and PTSD symptoms (YSRF), childhood trauma (CTQ) and violence exposure (SAVE). Functional neuroimaging of emotional responding to aversive stimuli in amygdala, hippocampus, dorsal and rostral anterior cingulate cortex, and ventromedial prefrontal cortex (BOLD fMRI signal during IAPS task)	Bilateral amygdala activity in response to aversive images was positively associated with attack-related PTS symptom severity in models controlling for internalising symptoms and prior violence exposure.
Moser et al. (2023)	Prospective case-control study of mothers exposed to domestic violence and their children conducted in Switzerland	Total *n* = 62 Mothers with significant PTSD symptoms *n* = 40 dyads Mothers without significant PTSD symptoms *n* = 22 dyads	Phase 1 Children 27.0 (8.6) months Range: 1–3.5 years Phase 2 Children 84.0 (12.9) months Range: 5–9 years	44%	Not reported	To determine if maternal psychopathology measured when children were in early childhood (phase 1) was associated with child psychopathology measured when children were of school-age (phase 2)	Phase 2 Number of PTSD symptoms (KSADS)	Phase 1 Socioeconomic status, child sex, and maternal child abuse history and parental behaviours, skills, and tendencies. Maternal measures of psychopathology included PTSD (CAPS, PCL-S), depression (BDI), dissociation (HDSC), alexithymia (TAS) and drug or alcohol use disorder. Phase 2 Child psychopathology including symptoms of depression, anxiety, ADHD, and behavioural disorders (KSADS). Child emotion comprehension (TfEC), bullying (SLS), exposure to violence or trauma (VEX-R), and representation of their parents and their emotions (MSSB)	Sparse canonical correlation analysis identified seven maternal factors at phase 1 and seven child outcome measures at phase 2. The highest weighted factor at phase 1 was maternal PTSD status and at phase 2 was child PTSD symptom scores.
Nixon et al. (2010)	Prospective study of children and adolescents recruited from two Australian hospital ED or paediatric inpatient wards following a single- trauma event	Total *n* = 48	11.84 (2.67) Range: 7–17	31%	Caucasian (88%), Other (12%)	To investigate the impact of cognitive (trauma-related appraisal) and biological factors (morphine usage and HR) as predictors of PTSD following a single-incident trauma	Within 4 weeks of trauma and at 6 months PTSD symptom severity (CPSS)	Morphine and paracetamol use and HR at triage Within 4 weeks of trauma Prior trauma history and treatment for emotional problems, injury severity, family history of psychological problems, pain at interview (FPRS-R), fear at the time of trauma (ASC-Kids), unhelpful appraisals (CPTCI) Within 4 weeks of trauma and at 6 months Depressive symptoms (CDI)	Fear at time of trauma, HR at triage, pain at interview, current depressive symptoms and unhelpful appraisals were positively correlated with PTSD symptoms at 4 weeks. Pain at interview, paracetamol dose, 4-week PTSD symptoms, and both 4-week and 6-month unhelpful appraisals and depressive symptoms predicted 6-month PTSD symptom severity. Morphine was associated with a reduction in PTSD symptoms over time.
Nugent et al. (2006)	Prospective cohort study of children and their primary caretakers recruited from a Midwestern (USA) hospital Emergency Department following paediatric injury	82 children and their primary caretakers (predomi-nantly mothers)	13.19 (2.94) Range: 8.04–17.89	Not specified	Caucasian (79.2%), African American (19.5%), mixed heritage (1.3%)	To identify whether parent PTS symptom severity is (1) associated with child PTS symptom severity and (2) moderates the relationship between child physiological measures collected within 12 hours of trauma and child 6-month PTS symptom scores	6 weeks & 6 months Child PTS symptom severity (CAPS-CA)	Child age, gender, race, type of trauma exposure, injury severity (ISS) and parental income Baseline First 20 min of HR data recorded during EMS transport and at ED admission and 12-hr urinary cortisol levels upon ED admission 6 weeks Parental distress (SCL-90-R) 6 weeks and 6 months Parental PTS symptoms (IES-R)	Higher child 6-week PTS scores were associated with lower parental income and child female sex. Higher parent 6-week PTS positively predicted child PTS at 6 weeks and 6 months. Low urinary cortisol and HR at hospitalisation predicted higher 6-month PTS scores in children with high parental PTS scores at 6 weeks.
Ostrowski et al. (2007)	Longitudinal analysis of children and their biological mothers recruited from an emergency department in the USA following paediatric injury	Baseline *n* = 54 mother-child dyads 6 weeks *n* = 45 mother-child dyads 7 months *n* = 38 mother-child dyads	13.35 (2.99)	24%	Not reported	To examine whether urinary cortisol levels collected from children within 12 hours of hospitalisation predicted 6-week and 7-month PTS symptom severity	6 weeks and 7 months Child PTS symptoms (CAPS-CA)	Baseline Child injury severity (ISS) and urinary cortisol as well as family income, child gender, age, race, parental education 6 weeks Child urinary cortisol 6 weeks and 7 months Child depressive symptoms (CDI) and maternal PTSD (CAPS) and depressive (CES-D) symptoms	Six-week PTS severity was higher in girls than in boys. Lower family income across participants and younger age in boys predicted higher 7-month PTS symptom severity. In models controlling for depressive symptoms, urinary cortisol at hospitalisation positively predicted 7-month PTS symptom severity in boys without a history of trauma exposure.
Ostrowski et al. (2006)	Longitudinal analysis of children and their biological mothers recruited from an emergency department in the USA following paediatric injury	Baseline *n* = 61 Follow-up *n* = 41	Males 13.35 (2.81) Females 13.29 (3.35) Range: 8–18	Baseline 45.90%	Baseline Caucasian (92%), African American (8%)	To investigate the influence of maternal PTS symptoms and child gender on child PTS symptoms 6 weeks and 7 months following traumatic injury	6 weeks and 7 months Child PTS symptom severity (CAPS-CA)	Baseline Child age, gender, race, and injury severity (ISS), as well as family income and parental education 6 weeks and 7 months Maternal PTSD symptoms (CAPS)	Six-week PTS scores were higher in females and were predicted by maternal PTS in males. Higher 7-month child PTS scores were associated with lower family income and higher maternal PTS across child sexes and by younger age in males. Higher maternal 6-week maternal PTS scores predicted 7-month PTS symptom severity in analyses excluding dyads where the mother was also a victim of the trauma.
Pervanidou, Kolaitis, Charitaki, Lazaropoulou, et al. (2007)	Prospective study of children and adolescents hospitalised after an MVA and unexposed controls in Greece	With MVA exposure Baseline *n* = 60 1 month *n* = 56 6 months *n* = 48 Controls *n* = 40	With MVA exposure at 6 months: 10.96 (2.51) Range: 7–18	With MVA exposure: 33.3%	White (100%) (reported in referenced study)	To investigate the influence of HPA axis, SNS and the inflammatory marker IL-6 on PTSD development following MVA exposure	1 and 6 months PTSD diagnosis (K-SADS- PL) and symptom severity (CPTR-RI)	Age, gender, race, injury severity (ISS), pubertal stage and BMI Baseline, 1 & 6 months Cortisol in saliva (collected at 08h00, 12h00, 15h00, 18h00 and 21h00), morning serum IL-6 and cortisol and plasma catecholamines	Morning serum IL-6 levels at hospitalisation were significantly higher in participants who met diagnostic criteria for PTSD at both 1- and 6-months post MVA. When controlling for morning serum cortisol, higher evening salivary cortisol and morning serum IL-6 at hospitalisation predicted 6-month PTSD diagnosis.
Pervanidou, Kolaitis, Charitaki, Margeli, et al. (2007)	Prospective study of children and adolescents hospitalised after an MVA and unexposed controls in Greece	With MVA exposure Baseline *n* = 60 1 month *n* = 56 6 months *n* = 48 Controls *n* = 40	With MVA exposure at baseline 10.7 (2.5) Range: 7–18	With MVA exposure 33.3%	White (100%)	To investigate whether HPA axis, SNS, and inflammatory markers after MVA predict PTSD at 1- and 6-months post MVA exposure	1 and 6 months PTSD diagnosis (K-SADS- PL) and symptom severity (CPTR-RI)	Age, gender, race, injury severity (ISS), pubertal stage and BMI Baseline, 1 & 6 months Salivary cortisol (collected at 08h00, 12h00, 15h00, 18h00 and 21h00), morning serum IL-6 and cortisol, and plasma catecholamines	Night salivary cortisol concentrations immediately after MVA, as well as 1- and 6-month plasma noradrenaline levels, were higher in participants with persistent (1 and 6 month) PTSD compared to MVA-exposed controls who did not meet PTSD criteria at either time point. Compared to MVA-exposed and non-exposed controls, 6-month plasma noradrenaline was higher in participants diagnosed with PTSD at 6 months and noon, evening and night salivary cortisol at hospitalisation was higher in participants diagnosed with PTSD at 1 and 6 months.
Pfeffer et al. (2007)	Prospective study of children with and without parental bereavement due to September 11, 2021, New York terror attack in the USA	Bereaved *n* = 45 Non-bereaved *n* = 34	Bereaved 8.9 (2.9) Range: 4.8–13 Non bereaved 9.3 (2.5) Range: 4.8–13.8	Bereaved 48.9% Non-bereaved 55.9%	Bereaved White (82.2%), Black (0%), Hispanic (4.4%), Asian (6.7%), mixed race (6.7%) Non-bereaved White (64.8%), Black (14.7%), Asian (2.9%), mixed race (8.8%)	To investigate the longitudinal relationship between markers of HPA axis activity and PTSD diagnosis following parental loss	6–monthly for 2 years PTSD diagnosis (K-SADS)	6-monthly for 2 years Morning and evening salivary cortisol collected over 3 days & following a dexamethasone suppression test	In samples collected at 16h00, cortisol levels were lower and cortisol suppression higher in bereaved participants with PTSD, regardless of comorbidity, compared to bereaved participants without psychiatric disorders and non-bereaved control participants.
Schneider and Gudiño (2018)	Longitudinal study of Latino adolescents at-risk for violence exposure recruited from a high school in Southern California (USA)	Baseline *n* = 168 6 months *n* = 161	11.4 (0.7) Range: 11–15	56.3%	Latino-Americans (100%), of which born outside of USA (36.9%)	To examine main and interaction effects of Latino cultural values and behavioural inhibition on the development of PTSD avoidance symptoms in adolescents at risk for violence exposure	Baseline & 6 months PTSD avoidance symptoms (CPSS)	Baseline Demographic variables (age, gender, school grade, racial/ethnic background, birthplace), Latino cultural values (MACVS), exposure to community violence (Modified EVS) and behavioural inhibition (BIS/BAS scale)	Violence exposure and behavioural inhibition were positively correlated with baseline and 6-month avoidance symptoms. The positive relationship between violence exposure and 6-month avoidance symptoms was stronger at higher levels of behavioural inhibition. Stronger Latino cultural values predicted a stronger positive relationship between behavioural inhibition and 6-month avoidance symptoms.
Smeeth et al. (2023)	Longitudinal study of Syrian refugee children and adolescents recruited in Lebanon to the Biological Pathways of Risk and Resilience in Syrian Refugee Children (BIOPATH) study	Baseline *n* = 1574 1 year *n* = 923	Baseline 11.4 (2.4) Range: 6–19 1 year 12.2 (2.4)	52.6%	Not reported	To investigate the relationships between war exposure, current living conditions, HCC, and PTSD symptomology in refugee children and adolescents	Baseline and 1 year PTSD symptom severity (CPSS)	Participant sex, nationality, current age, age at the end of war exposure, time since leaving Syria, smoking status, pubertal development, BMI, recent illness and endocrinological illness or medication Baseline War event exposure (WEQ) Baseline and 1 year HCC and current living conditions assessment (PREI)	The number of war-related events experienced was positively associated PTSD symptom severity, particularly in participants with an older age (≥ 12 years) at the time that war exposure ended. HCC was positively associated with PTSD symptom severity, and partially mediated the relationship between war event exposure and PTSD symptoms
Straub et al. (2017)	Longitudinal study of children and adolescents recruited in Germany to one of two studies examining (1) participants who recently experienced or witnessed an accident and (2) participants presenting with PTS following a previous trauma	Study 1 *n* = 35 Study 2 *n* = 22	Study 1 10.71 (2.96) Study 2 12.1 (3.4)	Study 1 34.3% Study 2 50.0%	Not reported	To examine whether (1) HCC differs between participants with and without PTS symptoms (study 1 & 2), (2) pre-trauma predicts development of PTS symptoms (study 1) and (3) HCC is correlated with PTS symptoms severity in a clinical sample	*Study 1* 1 week post trauma Acute stress symptoms (ASS) 6–8 weeks post trauma PTS symptom severity (CAPS-CA) *Study 2* 29.5 (22.7) months post trauma PTS symptom severity (CAPS-CA)	Study 1 & 2 Demographic (age, sex, nationality), hair (natural hair colour, artificial hair colouring), trauma-related (type, multiple trauma experience, injury severity), BMI and smoking and pharmaceutical or illegal drug use variables Study 1 1 week post trauma: HCC Study 2: 29.5 (22.7) month post trauma: HCC	The HCC of patients with PTS were lower than the HCC of healthy controls (study 1 + 2). Group and group-sex interaction findings indicate lower HCC in participants with PTS and particularly in male participants. Lower pre-trauma HCC predicted 1-week dissociation and 6–8-week hyperarousal symptoms in females. HCC was inversely associated with hyperarousal symptoms in participants with chronic PTS.
Tang et al. (2018)	Longitudinal cohort study comprising children recruited from child protection agencies in Ontario, Canada.	*n* = 43	14.07 (1.13) Range: 12–16	100%	Not reported	To ascertain whether consistent intra-individual patterns of frontal asymmetry act as a moderator of the childhood maltreatment – PTSD symptom relationship	Baseline, 6, 12, 18 and 24 months PTSD symptoms or disorder (KSADS-E)	Baseline Age, Tanner pubertal stage, handedness, child protection agency adjudications, childhood trauma (CTQ and CEVQ) and current and past 6-month psychiatric disorder (KSADS-E) Three measurements over 2 years Resting frontal EEG alpha asymmetry	Current PTSD symptoms over 2 years was positively related to more advanced pubertal stage at baseline and the interaction of frontal alpha asymmetry profiles and childhood trauma severity. At lower levels of trauma exposure, participants with a stable right asymmetry profile were more likely to report current symptoms and/or episodes of PTSD over the two-year study period.
Vasa et al. (2004)	Prospective cohort study investigating neuroimaging correlates of mental health outcomes following TBI in children and adolescents in the USA	*n* = 95	10.62 (3.85) Range: 4–19	43%	African American (54%), Caucasian/other (46%)	To examine whether the volume and number of lesions in five brain regions are associated with total and domain PTS severity	Baseline and 1 year PTSD diagnosis and symptom severity (DICA)	Baseline Injury severity (GCS) and psychosocial adversity (Modified Psychosocial Adversity Scale) 3 months MRI measures of the number and volume of lesions in the mesial and dorsolateral prefrontal cortex, orbitofrontal cortex, temporal lobe, and diffuse brain (nonprefrontal and nontemporal) Baseline and 1 year Anxiety disorder diagnosis and symptom severity (DICA)	Higher PTS and hyperarousal symptom scores were associated with presence and volume of left temporal lobe lesions and absence of left orbital lesions. Presence of a left orbitofrontal cortex lesion predicted lower hyperarousal symptoms and the number of diffuse lesions was positively associated with avoidance symptoms.

*Data refer to age at baseline unless otherwise stated. ASC-Kids: Acute Stress Checklist for Children; BDI: Beck Depression Injury; BIS/BAS: Behavioural Inhibition System/Behavioural Activation System Scale; BMI: body mass index; BSI: Brief Symptom Inventory; BSQ: Behavioural Systems Questionnaire; CAPS: Clinician-Administered PTSD Scale, CAPS-CA: Clinician–Administered PTSD Scale for Children and Adolescents; CDI: Children’s Depression Inventory; CEVQ: Childhood Experiences of Violence Questionnaire; CMS: Children’s Memory Scale; CNT: Children’s Naming Test; CPSS: Child PTSD Symptom Scale; CPTCI: Child Post-Traumatic Cognitions Inventory; CPT-RI: Children’s Posttraumatic Reaction Index; CT: computerised tomography; CTQ: Childhood Trauma Questionnaire; EVS: Exposure to Violence Scale; GCS: Glasgow Coma Scale; FPRS-R: Faces Pain Rating Scale; HCC: hair cortisol concentration; HDSC: Hopkins Dissociative Symptoms Checklist; HICUPS: How I Coped Under Pressure Scale (HICUPS); IES-R: Impact of Events Scale-Revised; KSADS: Kiddie Schedule for Affective Disorders and Schizophrenia; KSADS-E: Schedule for Affective Disorders and Schizophrenia for School-Aged Children-Epidemiologic Version 5; K-SADS-PL: Children’s Schedule for Affective Disorders and Schizophrenia-present and lifetime version for children; MACVS: Mexican American Cultural Values Scale; MASC: Multidimensional Anxiety Scale for Children; MFQ: Mood and Feelings Questionnaire; MRI: magnetic resonance imaging; MSSB: MacArthur Story Stem Battery; PCL-S: Posttraumatic Stress Disorder Checklist Scale; PREI: Perceived Refugee Environment Index; PTSD-RI: University of California PTSD Reaction Index; RSA: Respiratory Sinus Arrhythmia; SAVE: Screen for Adolescent Violence Exposure; SCARED: Screen for Child Anxiety Related Emotional Disorders; SCL-90-R: Symptom Checklist-90-R; SLES: Stressful Life Events Schedule Adolescent Report; SLS: School-Life Survey; TAS: Toronto Alexithymia Scale; TEA-Ch: Test of Everyday Attention for Children; TfEC: Test for Emotional Comprehension; VEX-R: Violence Exposure Scale – Revised; WASI: Weschler Abbreviated Scale of Intelligence; WEQ: War Events Questionnaire; WISC-IV DSb: Weschler Intelligence Scale for Children, fourth edition, Digit Span backwards; YSR: Youth Self Report Form

### 2.4. Quality assessment

The Systematic Appraisal of Quality in Observational Research rubric, which was designed to evaluate the quality of evidence-based cohort and case–control observational studies in psychiatric research, was used to score manuscripts across six categories: sample, control/comparison group, measurement and output quality, follow-up, distorting influences, and data reporting (Ross et al. 2011). Each category comprises between two and five statements, which can be scored as adequate, unclear, inadequate, or not applicable, with the sum of these decisions used to designate the study quality (Table 2). The quality assessments were performed by different coauthors, and then collated and reviewed by the senior author to ensure accuracy and consistency of the scoring.

**Table 2.** Assessment of study quality according to the systematic appraisal of quality in observational research tool.

Study	Sample	Control/comparison group	Exposure/predictors and outcome measures	Follow-up	Distorting influences	Reporting of data	Overall study quality
Delahanty et al. (2005)	Children admitted to hospital following traumatic injuries. Sample source and inclusion/exclusion criteria are clear. The sampling method, representativeness of participants and adequacy of sample size are unclear. *(Quality: unclear)*	N/A	Exposure/predictors Injury severity and urinary catecholamine and cortisol levels following traumatic injury exposure. Outcome PTSD symptoms Exposure and outcome measures were adequately assessed. *(Quality: adequate)*	The number of participants lost to follow-up and the reasons for loss are clear. *(Quality: adequate)*	Age, race, gender, depression and parental education and income were considered. *(Quality: adequate)*	Data are clearly reported. Sample sizes are reported for each analysis but variation in sample size is not explained. No missing data statement provided. *(Quality: unclear)*	Moderate
Geng et al. (2019)	Adolescent survivors who participated in the Wenchuan Earthquake Adolescent Health Cohort (WEAHC) study. Sampling source, sampling method, and inclusion/exclusion criteria are clear, and the sample size is adequate for the analyses performed. Referenced paper provides information on representativeness. *(Quality adequate)*	N/A	Exposure/predictors Insomnia and depression symptoms following earthquake exposure. Outcome PTSD symptom Exposure and outcome measures were adequately assessed. *(Quality: adequate)*	The number of participants lost to follow-up and the reasons for loss are clear. *(Quality: adequate)*	Demographic and earthquake exposure variables were considered. *(Quality: adequate)*	Data are clearly reported. Missing data were replaced with estimates derived by single imputation. Analyses including and excluding participants with imputed data produced similar results. *(Quality: adequate)*	Very high
Guo et al. (2017)	Children admitted to hospital following TBI. Sample source and inclusion/exclusion criteria are clear and the sample size is adequate. Sample representativeness and sampling method are unclear. *(Quality: adequate)*	N/A	Exposure/predictors TBI variables and neurocognitive measures Outcome PTSD symptom severity Exposure and outcome measures were adequately assessed. *(Quality: adequate)*	The number of participants lost to follow-up is stated but explanations for loss are not provided. *(Quality: unclear)*	Sex, socioeconomic status, IQ, age at injury and injury severity were considered. *(Quality: adequate)*	Data are clearly reported. No missing data statement provided. *(Quality: unclear)*	Moderate
Haag et al. (2019)	Trauma exposed children and their parents recruited from four hospitals in the UK. Sample source and inclusion/exclusion criteria are clear and referenced paper provides sampling method details. The representativeness of participants and the adequacy of the sample size for longitudinal analyses are unclear. *(Quality: adequate)*	N/A	Exposure/predictors Trauma exposure variables as well as HR and HRV indices at rest and during child and joint parent-child narrative recount of trauma Outcome PTSD symptom severity Exposure and outcome measures were adequately assessed. *(Quality: adequate)*	The number of participants lost to follow-up and the reasons for loss are clear. *(Quality: adequate)*	Age, sex, and triage were considered. *(Quality: adequate)*	Data are clearly reported. No missing data statement provided. *(Quality: unclear)*	High
Heyn and Herringa (2019)	Youths recruited to the Youth PTSD Study in the USA. Sampling source, sampling method, and inclusion/exclusion criteria are clear, and the sample size is adequate for the analyses performed. Representativeness is unclear. *(Quality: adequate)*	Control group comprising typically developing youth was easily identifiable, matched to cases for age and sex and drawn from the community. *(Quality: adequate)*	Exposure / predictors Cortical thickness and surface area. Outcome PTSD case/control status and symptom severity. Exposure and outcome measures were adequately assessed. *(Quality: adequate)*	The number of participants lost to follow-up and the reasons for loss are clear. *(Quality: adequate)*	Age, sex, IQ, race, parental education, pubertal stage, history of psychotropic medication or therapy, and trauma variables were considered. *(Quality: adequate)*	Data are clearly reported with an explanation for missing data provided. *(Quality: adequate)*	Very high
Heyn et al. (2022)	Participants recruited to the Youth PTSD Study in the USA. Sampling source, sampling method, and inclusion/exclusion criteria are clear. Representativeness and adequacy of the sample size are unclear. *(Quality: adequate)*	N/A	Exposure / predictors Cortical surface area and voxel-based morphometry Outcome PTSD case-control status and symptom severity. Exposure and outcome measures were adequately assessed. *(Quality: adequate)*	Referenced paper provides information on sample attrition. *(Quality: adequate)*	Age, sex, IQ, pubertal stage, intracranial volume, childhood abuse severity, index trauma type, age at index trauma, trauma and stressful life event load, internalising disorder comorbidity, and history of psychotropic medication use and psychotherapy were considered. *(Quality: adequate)*	Data are clearly reported. No missing data statement provided. *(Quality: unclear)*	High
Kolaitis et al. (2011)	Sampling source, method, and inclusion/exclusion criteria are clear. Representativeness was unclear and the study had low power to detect differences. *(Quality: adequate)*	N/A	Exposure/predictors Road traffic accident exposure and salivary cortisol measured within 24 hours of the accident. Outcome PTSD diagnosis Exposure and outcome measures were adequately assessed. *(Quality: adequate)*	The number of participants lost to follow-up and the reasons for loss are clear. *(Quality: adequate)*	Age, gender, injury severity and brain injury severity considered. *(Quality: adequate)*	Data are clearly reported with an explanation for missing data provided. *(Quality: adequate)*	Very high
Linde-Krieger et al. (2024)	Sampling source, method, inclusion/exclusion criteria, representativeness and the adequacy of the sample size are clear. *(Quality: adequate)*	N/A	Exposure / predictors Respiratory sinus arrhythmia during childhood, caregiver internalising psychopathology and perceived caregiver attachment at early adolescence and PTSD symptom severity one-year prior to the pandemic. Outcome PTSD symptom severity during the COVID-19 pandemic. Exposure and outcome variables were adequately assessed. *(Quality: adequate)*	The number of participants with data at each time point is clear and reasons for loss to follow-up are provided. *(Quality: adequate)*	Pre-pandemic PTSD symptom severity, ethnicity/race, sex, and income-to-needs considered. *(Quality: adequate)*	Data are clearly presented. Missing data were estimated using the Full Information Maximum Likelihood approach. *(Quality: adequate)*	Very high
Luo et al. (2012)	Female adolescents with and without exposure to the Wenchuan earthquake in China. Sampling source, method, inclusion/exclusion criteria, and the adequacy of the sample size are clear. Representativeness is unclear. *(Quality: adequate)*	Control group comprising participants without earthquake exposure is easily identifiable and matched by age, sex, and education level. The source of the controls is clear, and group differences are controlled for using statistical measures. *(Quality: adequate)*	Exposure / predictors Experience of earthquake-related traumatic events and 7-month HCC reflecting four 3-month periods. Outcome PTSD symptom severity and diagnosis. Exposure and outcome variables were adequately assessed. *(Quality: adequate)*	No participants were lost to follow-up. *(Quality: adequate)*	Study recruited only female participants and were matched by age and education. *(Quality: adequate)*	Data are clearly presented. No missing data statement provided. *(Quality: unclear)*	High
Marsac et al. (2017)	Children admitted for treatment of an injury at a paediatric trauma centre in the northeastern USA. Sampling source, method, and inclusion/exclusion criteria are clear. Representativeness and the adequacy of the sample size are unclear. *(Quality: adequate)*	N/A	Exposure / predictors Injury severity, trauma history, global and trauma-specific appraisals, posttraumatic appraisals, coping and HR. Outcome PTS symptom severity Exposure and outcome measures are adequately assessed. *(Quality: adequate)*	The number of participants lost to follow-up and the reasons for loss are clear. *(Quality: adequate)*	Age, sex, injury severity, race/ethnicity, and baseline PTS symptoms were considered. *(Quality: adequate)*	Data are clearly presented. Missing data were estimated using a Full Information Maximum Likelihood approach. (*Quality: adequate*)	Very high
McLaughlin et al. (2014)	Adolescents who had previously completed an fMRI task measuring emotional responding and who had subsequently been indirectly exposed to the Boston Marathon attack. Sampling method clear. Sample source and inclusion/exclusion criteria unclear. The representativeness of the sample and the sample size are not adequate. *(Quality: inadequate)*	N/A	Exposure / predictors Pre-existing internalising symptoms, childhood trauma and violence exposure, brain region activity in response to aversive images Outcome PTS symptom severity Exposure and outcomes were adequately assessed. *(Quality: adequate)*	No participants were lost to follow-up. *(Quality: adequate)*	Gender, age, time since scan, pre-existing internalising symptoms and violence exposure considered. *(Quality: adequate)*	Data were clearly and accurately reported. No missing data statement provided. *(Quality: unclear)*	Moderate
Moser et al. (2023)	Domestic violence-exposed mothers and their infants recruited to the Geneva Early Childhood Stress Study. Sampling source, sampling method, inclusion/exclusion criteria and the adequacy of the sample size are clear. Representativeness is unclear. *(Quality: adequate)*	Control group comprising children born to mothers without PTSD is easily identifiable and the source is clear. There were no differences between cases and controls for maternal age, child age or sex, or socioeconomic status. *(Quality: adequate)*	Exposure/predictors Child psychopathology (bullying, emotion comprehension, representation of their parents and their emotions, and exposure to crime and trauma), socioeconomic status and child sex, as well as maternal psychopathology, parental behaviours, and child abuse experience. Outcome PTSD symptoms Exposure and outcomes were adequately assessed. *(Quality: adequate)*	The number of participants lost to follow-up and the reasons for loss are clear. *(Quality: adequate)*	Socioeconomic status and child sex were included in analyses *(Quality: adequate)*	Data were clearly reported and how missing data were managed was stated. *(Quality: adequate)*	Very high
Nixon et al. (2010)	Children and adolescents recruited from the emergency unit or paediatric ward following a single traumatic injury requiring hospitalisation. Sample source and inclusion/exclusion criteria clear and sampling method details provided in referenced paper. Adequacy of sample size and sample representativeness not clear. *(Quality: adequate)*	N/A	Exposure/predictors Single trauma event requiring hospitalisation. HR at triage morphine and non-opioid pain medication usage, fear at the time of trauma, injury severity, depressive symptoms and posttraumatic cognitions assessed. Outcome PTSD symptoms Exposure and outcome variables were adequately assessed. *(Quality: adequate)*	No participants lost to follow-up. *(Quality: adequate)*	Medication, fear at time of traumatic event, and demographic factors (unspecified) considered *(Quality: adequate)*	Data clearly reported. No missing data statement provided. *(Quality: unclear)*	High
Nugent et al. (2006)	Children and their primary caretakers recruited from a Midwestern Hospital’s emergency department following admission for paediatric injury. Sample source and sampling method are clear and referenced study details inclusion/exclusion criteria and representativeness. Adequacy of the sample size is unclear. *(Quality: adequate)*	N/A	Exposure / predictors Child HR and urinary cortisol and parental general distress and PTS symptom severity. Outcome Child PTS symptoms Exposure and outcome measures were adequately assessed. *(Quality: adequate)*	The number of participants lost to follow-up and the reasons for loss are clear. *(Quality: adequate)*	Child age, gender, race, trauma type, injury severity and parent income and education considered. *(Quality: adequate)*	Data are clearly reported. No missing data statement provided. *(Quality: unclear)*	High
Ostrowski et al. (2007)	Children and their biological mothers recruited from an Emergency Department in the USA following paediatric injury. Sample source, sampling method, and inclusion/exclusion criteria are clear. Sample representativeness and adequacy of the sample size are unclear. *(Quality: adequate)*	N/A	Exposure / predictors Trauma exposure requiring hospitalisation and child depressive symptoms. Outcome Child PTS symptom severity Exposure and outcome measures were adequately assessed. *(Quality: adequate)*	Number of participants lost to follow-up indicated but explanations for loss are not provided. *(Quality: unclear)*	Child sex, age, race, injury severity, and previous trauma experience, as well as parental education and family income were considered. *(Quality: adequate)*	Most data clearly reported. Missing data is mentioned but no explanation provided. *(Quality: unclear)*	Moderate
Ostrowski et al. (2006)	Children and their biological mothers recruited from an Emergency Department in the USA following paediatric injury. Sample source is clear. Inclusion/exclusion criteria, sampling method, representativeness and adequacy of sample size unclear. (Quality: inadequate)	N/A	Exposure / predictors Trauma exposure requiring hospitalisation and maternal 6-week and 7-month PTS symptom severity. Outcome Child PTS symptom severity Exposure and outcome variables were adequately assessed. *(Quality: adequate)*	Number of participants lost to follow-up indicated but explanations for loss are not provided. *(Quality: unclear)*	Child sex, age, race, and injury severity, as well as family income, parental education and maternal direct experience of traumatic incident were considered. *(Quality adequate)*	Most data clearly reported. No missing data statement provided. *(Quality: unclear)*	Moderate-low
Pervanidou, Kolaitis, Charitaki, Margeli, et al. (2007)	Children and adolescents with and without MVA-related exposure and injury. Sample source and inclusion/exclusion criteria clear. Sampling method detailed in referenced study. Representativeness and adequacy of sample size unclear. *(Quality: adequate)*	Control group clearly identified and matched for age and BMI. Referenced study details sample source. *(Quality: adequate)*	Exposure / predictors MVA, salivary cortisol, morning serum IL-6 and cortisol and plasma catecholamines Outcome PTSD diagnostic status and symptom severity Exposure and outcome variables were adequately assessed. *(Quality: adequate)*	Number lost to follow up stated with reasons provided in referenced paper. *(Quality: adequate)*	Age, BMI, sex, race, pubertal stage, and injury severity were considered. *(Quality: adequate)*	Most data clearly reported. No missing data statement provided. *(Quality: unclear)*	High
Pervanidou, Kolaitis, Charitaki, Lazaropoulou, et al. (2007)	Children and adolescents with and without MVA-related exposure and injury. Sample source, inclusion/exclusion criteria and sampling method clear. Representativeness and adequacy of sample size unclear. *(Quality: adequate)*	Control group comprising siblings of children attending an obesity clinic is clearly identified and matched for age and BMI. Control group source is clear. *(Quality: adequate)*	Exposure / predictors MVA, salivary cortisol, morning serum and cortisol, and plasma catecholamines Outcome PTSD diagnostic status and symptom severity Exposure and outcome variables were adequately assessed. *(Quality: adequate)*	Number lost to follow up stated and explanation provided. *(Quality: adequate)*	Age, BMI, sex, and injury severity were considered. *(Quality: adequate)*	Most data clearly reported. Missing data handled by repeating analysis excluding participants with missing data. *(Quality: adequate)*	Very high
Pfeffer et al. (2007)	Bereaved and non-bereaved children in the New York City metropolitan area Sample source is clear. Inclusion/exclusion criteria, representativeness, sampling method and adequacy of sample size are unclear. *(Quality: inadequate)*	Control group comprising non-bereaved children is easily identified and matched for age, gender, ethnicity, BMI, and family status and lifetime stressful events prior to trauma. Control group source is clear. *(Quality: adequate)*	Exposure/predictors Loss of a parent, and morning and afternoon salivary cortisol. Outcome PTSD diagnosis Exposure and outcome variables were adequately assessed. *(Quality: adequate)*	Number of participants lost to follow- up stated and explanation provided. *(Quality: adequate)*	Age, gender, ethnicity, and BMI were considered. *(Quality: adequate)*	Data are clearly reported with an explanation for missing data provided. *(Quality: adequate)*	High
Schneider and Gudiño (2018)	Latino students recruited from a large middle school in Southern California. Sample source and representativeness, and sampling method clear. Inclusion/exclusion criteria and adequacy of sample size not clear. *(Quality: adequate)*	N/A	Exposure / predictors Cultural values, exposure to violence and behavioural inhibition Outcome PTSD avoidance symptoms Exposure and outcome variables were adequately assessed. *(Quality: adequate)*	Number of participants lost to follow up clear but explanation for loss not provided. *(Quality: adequate)*	Sex, age, baseline PTSD avoidance symptoms and classroom clustering considered. *(Quality: adequate)*	Data were clearly reported with missing data handled via list-wise deletion. *(Quality: adequate)*	Very high
Smeeth et al. (2023)	Syrian children and adolescents living in refugee camps in Lebanon. Sample source, sampling method, and inclusion/exclusion criteria are clear, and the sample size is adequate for the analyses performed. Representativeness is unclear. *(Quality: adequate)*	N/A	Exposure / predictors HCC, exposure to war-related events and perceived quality of the refugee environment Outcome PTSD symptom severity Exposure and outcome variables were adequately assessed. *(Quality: adequate)*	The number of participants lost to follow-up and the reasons for loss are clear. *(Quality: adequate)*	Age, sex, pubertal stage, BMI, nationality, smoking status, as well as hair washing, hair treatment, month of hair collection and analysis batch were considered. *(Quality: adequate)*	Data are clearly reported with an explanation for missing data provided. *(Quality: adequate)*	Very high
Straub et al. (2017)	Children and adolescents recruited from hospital wards within 1 week of experiencing or witnessing a trauma or from an outpatient psychiatry and psychotherapy clinic months to years following the most recent traumatic event. Sample source, sampling method and inclusion/exclusion criteria are clear. Adequacy of sample size and representativeness are unclear. *(Quality: adequate)*	Participants recruited within 1 week of trauma exposure designated as healthy controls for the purpose of HCC comparisons. Identification and source of control group are clear. Groups did not differ according to age, sex, BMI, or medication use. *(Quality: adequate)*	Exposure / predictors Witnessing or experiencing trauma and HCC Outcome*s* PTS total and hyperarousal, re-experiencing, and avoidance domain symptom scores. Exposure and outcome variables were adequately assessed. *(Quality: adequate)*	No participants were lost to follow up. *(Quality: adequate)*	Age, sex, BMI, smoking status, pharmaceutical and illegal drug use, as well as artificial hair colouring and number of hair washes/week were considered. *(Quality: adequate)*	Data were clearly and accurately reported. No missing data statement provided. *(Quality: unclear)*	High
Tang et al. (2018)	Maltreated adolescents recruited from child protection agencies in Ontario, Canada. Sample source, sampling method, and inclusion/exclusion criteria are clear. The adequacy of the sample size and representativeness of the sample are unclear. *(Quality: adequate)*	NA	Exposure / predictors Self-report and child protection agency assessment of childhood trauma exposure, as well as depressive symptom severity and resting frontal EEG alpha asymmetry. Outcome*s* PTSD symptom severity Exposure and outcome measures were adequately assessed. *(Quality: adequate)*	The number of participants lost to follow-up and the reasons for loss are clear. *(Quality: adequate)*	Initial age of the adolescents, Tanner pubertal stage, and handedness were considered. *(Quality: adequate)*	Data are clearly presented. Missing data handled via linear interpolation imputation. *(Quality: adequate)*	Very high
Vasa et al. (2004)	Children and adolescents with severe TBI recruited from a rehabilitation centre for children with neurological disorders in the USA. Inclusion/exclusion criteria clear and referenced papers provide information on sample source and sampling method. Representativeness of the sample and adequacy of the sample size are unclear. *(Quality: adequate)*	N/A	Exposure / predictors Moderate or severe closed head injury. Neuroimaging measures of brain volumes and lesions Outcome Total PTS, reexperiencing, hyperarousal and avoidance symptom scores. Exposure and outcome variables were adequately assessed. *(Quality: adequate)*	No participants were lost to follow-up. *(Quality: adequate)*	Age, gender, whole brain volume, psychosocial adversity score, preinjury anxiety symptoms and injury severity were considered. *(Quality: adequate)*	Data are clearly reported. No missing data statement provided. *(Quality: unclear)*	High

HCC: hair cortisol concentration; PTSD: posttraumatic stress disorder.

## 3. Results

### 3.1. Description of studies

Twenty-four longitudinal studies, covering a range of designs, settings, and sample characteristics, were included. The studies investigated the development and trajectories of PTSD symptoms in children and adolescents following physical injury (*n* = 8), being involved in or witnessing accidents (*n* = 6), natural disasters (*n* = 2), exposure to domestic violence (*n* = 2) and terrorist attacks (*n* = 2), as well as community violence, child maltreatment, conflict and the COVID-19 pandemic (*n* = 1 each) (Delahanty et al., 2005; Geng et al., 2019; Guo et al., 2017; Haag et al., 2019; Heyn et al., 2022; Heyn & Herringa, 2019; Kolaitis et al., 2011; Linde-Krieger et al., 2024; Luo et al., 2012; Marsac et al., 2017; McLaughlin et al., 2014; Moser et al., 2023; Nixon et al., 2010; Nugent et al., 2006; Ostrowski et al., 2006; Ostrowski et al., 2007; Pervanidou, Kolaitis, Charitaki, Margeli, et al., 2007; Pervanidou, Kolaitis, Charitaki, Lazaropoulou, et al., 2007; Pfeffer et al., 2007; Schneider & Gudiño, 2018; Smeeth et al., 2023; Straub et al., 2017; Tang et al., 2018; Vasa et al., 2004) The studies ranged from 6 weeks to 36 months post-trauma and were conducted in various settings, including hospitals, schools, and communities. Half of the studies (*n* = 12) were conducted in the USA. Three studies were performed in Greece (Kolaitis et al., 2011; Pervanidou, Kolaitis, Charitaki, Margeli, et al., 2007; Pervanidou, Kolaitis, Charitaki, Lazaropoulou, et al., 2007), two studies were performed in Australia (Guo et al., 2017; Nixon et al., 2010), and one study was performed in each of Canada (Tang et al., 2018), Germany (Straub et al., 2017), Switzerland (Moser et al., 2023) and the United Kingdom (Haag et al., 2019). Only three studies were conducted in low- or middle-income countries (LMICs) (China, *n* = 2 [Geng et al., 2019; Luo et al., 2012] and Lebanon, *n* = 1 [Smeeth et al., 2023]) and none of the identified studies were conducted in South America or Africa. The sample sizes ranged from relatively small (*n* = 15)(McLaughlin et al., 2014) to larger cohorts (*n* = 1574 at baseline) (Smeeth et al., 2023). The majority of studies specified participants’ ages at baseline assessment, with these ages ranging from 4 to 19 years. Six studies also included parental or caregiver assessments (Kolaitis et al., 2011; Linde-Krieger et al., 2024; Moser et al., 2023; Nugent et al., 2006; Ostrowski et al., 2006; Ostrowski et al., 2007). The samples were diverse in terms of sex, and most of those that reported on ancestry or ethnicity reflected local population demographics, although some studies focussed on specific populations, such as Latino adolescents (Schneider & Gudiño, 2018) or Syrian refugee children (Smeeth et al., 2023). A range of clinical measures were used to assess PTSD diagnosis and symptoms with six studies employing multiple measures (Heyn et al., 2022; Heyn & Herringa, 2019; Kolaitis et al., 2011; Luo et al., 2012; Pervanidou, Kolaitis, Charitaki, Margeli, et al., 2007; Pervanidou, Kolaitis, Charitaki, Lazaropoulou, et al., 2007). The most frequently used measures were the child/adolescent version of the Clinician-Administered PTSD Scale for DSM-5 (*n* = 6) and the Child PTSD Symptom Scale (*n* = 4).

### 3.2. Risk of bias and limitations in included studies

The overall quality of most studies was rated as high (Geng et al., 2019; Haag et al., 2019; Heyn et al., 2022; Heyn & Herringa, 2019; Kolaitis et al., 2011; Linde-Krieger et al., 2024; Luo et al., 2012; Marsac et al., 2017; Moser et al., 2023; Nixon et al., 2010; Nugent et al., 2006; Pervanidou, Kolaitis, Charitaki, Lazaropoulou, et al., 2007; Pervanidou, Kolaitis, Charitaki, Margeli, et al., 2007; Pfeffer et al., 2007; Schneider & Gudiño, 2018; Smeeth et al., 2023; Straub et al., 2017; Tang et al., 2018; Vasa et al., 2004), with adequate scores for at least four of the six categories assessed. However, over half of the studies did not report data or missing data clearly and many did not adequately address sample size or representativeness, which can affect generalisability and reliability. Several studies were rated moderate or moderate-to-low in quality (Delahanty et al., 2005; Guo et al., 2017; McLaughlin et al., 2014; Ostrowski et al., 2006; Ostrowski et al., 2007) due to sampling, representativeness or missing data issues. Many studies had small sample sizes (*n* = 19, < 100), increasing bias risk and limiting generalisability. Several studies relied solely on self-report measures (*n* = 7), which are prone to recall and response biases. Different diagnostic criteria and assessment tools limited direct comparisons. Varied follow-up periods made it difficult to establish causality or examine long-term outcomes. Although all studies were longitudinal, not all studies assessed PTS symptoms and all biopsychosocial variables at every study time point, limiting predictive power. Some studies did not account for confounding variables like age, education, income, lifestyle, medication use, or comorbid conditions. Most studies focussed on a single traumatic event, limiting generalisability to those with multiple trauma exposures. Additionally, not all studies specified whether participants experienced or witnessed trauma and responses to different types or numbers of traumas were not compared. Only seven studies (Heyn & Herringa, 2019; Luo et al., 2012; Moser et al., 2023; Pervanidou, Kolaitis, Charitaki, Lazaropoulou, et al., 2007; Pervanidou, Kolaitis, Charitaki, Margeli, et al., 2007; Pfeffer et al., 2007; Straub et al., 2017) included control groups, making it difficult to determine if observations were solely related to PTSD symptom trajectory or also influenced by trauma exposure effects.

### 3.3. Synthesis of results

#### 3.3.1. Biological factors

The range of biological factors investigated can be broadly categorised as measures of HPA axis and autonomic nervous system (both sympathetic and parasympathetic) activity, as well as structural brain morphology and functional neuroimaging investigations. Of these, cortisol, the primary glucocorticoid and effector of the HPA-axis was the most commonly investigated stress marker. Several studies found that acute and chronic cortisol levels were associated with PTSD symptoms, although the direction of the association varied according to the source material, type of trauma and the time elapsed since the trauma (Heyn & Herringa, 2019; Kolaitis et al., 2011; Luo et al., 2012; Nugent et al., 2006; Pervanidou, Kolaitis, Charitaki, Margeli, et al., 2007; Pervanidou, Kolaitis, Charitaki, Lazaropoulou, et al., 2007; Pfeffer et al., 2007; Smeeth et al., 2023; Straub et al., 2017). Hair cortisol concentrations (HCC), reflecting the past three-month cortisol levels, were positively associated with PTSD symptom severity at baseline and after one year in conflict-exposed Syrian refugee children and adolescents (mean age 11.4 years) (Smeeth et al., 2023). However, HCC was lower at two-to-four and five-to-seven months post-earthquake exposure in female adolescents (age range of 12–15 years) diagnosed with PTSD seven months after the earthquake (Luo et al., 2012) and was found to be inversely associated with posttraumatic stress (PTS) symptom severity in a sex-specific manner in a study of children and adolescents (mean age 10.7 years) hospitalised for traumatic injuries (Straub et al., 2017). Specifically, lower pre-trauma HCC predicted one-week dissociation and six–to-eight-week hyperarousal symptoms in females. Urinary cortisol levels in the acute aftermath of traumatic injury were higher in children and adolescents (mean age 13.0 years) with worse PTS symptoms at six weeks follow up (Delahanty et al., 2005) and in children and adolescents (mean age 13.4 years) with worse symptoms at seven months post injury (Ostrowski et al., 2007), though the latter finding only held for males. Interestingly, in a study of children and adolescents (mean age 13.3 years) hospitalised following exposure to a variety of traumatic events, lower urinary cortisol at hospitalisation was associated with higher six-month PTS severity but only in children whose parents also showed concurrent elevated PTS scores (Nugent et al., 2006). Studies found that higher evening salivary cortisol in youth (6–18 years of age) admitted to hospital following a motor vehicle accident (MVA) predicted PTSD diagnosis at one and six months (Kolaitis et al., 2011; Pervanidou, Kolaitis, Charitaki, Margeli, et al., 2007; Pervanidou, Kolaitis, Charitaki, Lazaropoulou, et al., 2007). However, in a study of psychopathology in children (4.8–13.8 years of age) who suffered loss of a parent in the September 11 2001 terrorism attack, bereaved children with PTSD showed lower afternoon salivary cortisol levels and increased cortisol sensitivity across the 18-month study period compared to trauma-exposed controls (Pfeffer et al., 2007).

Several studies investigated clinical and molecular markers of autonomic nervous system activity. With respect to sympathetic nervous activity, the ‘fight or flight’ response activated in response to acute threat, urinary epinephrine measured within 12 hours of children and adolescents (mean age 13.0 years) being hospitalised for traumatic injury was positively associated with PTSD symptoms six weeks later (Delahanty et al., 2005). Plasma noradrenaline levels in children and adolescents (mean age 10.7 years) measured at one- and six-months after MVA were positively associated with PTSD persistence, i.e. meeting criteria for PTSD diagnosis at both time points (Pervanidou, Kolaitis, Charitaki, Lazaropoulou, et al., 2007). Higher morning serum concentration of the pro-inflammatory cytokine interleukin-6 at hospitalisation was also positively associated with PTSD persistence in the same study group (Pervanidou, Kolaitis, Charitaki, Margeli, et al., 2007). Studies examining the relationship between PTSD and heart rate (HR), which can reflect sympathetic tone, revealed discrepant results. In studies assessing HR at hospitalisation following traumatic injury, Marsac et al. (2017) found no HR association with posttraumatic stress severity at six or 12 weeks in participants (mean age 10.6 years), while Nixon et al. (2010) found a positive correlation between HR at triage and PTSD symptoms at four weeks but not six months in children and adolescents (mean age 11.8 years). In a study of participants (mean age 13.3 years) with traumatic injuries, the inverse association between HR at hospitalisation and six-week PTS severity was contingent on parents reporting higher concurrent PTS scores (Nugent et al., 2006). In a study of children (mean age 10.1 years) hospitalised following acute trauma, a higher mean change in HR assessed when children and parents jointly recounted the trauma one month following exposure predicted more severe PTSD symptomology at three months post trauma (Haag et al., 2019).

Parasympathetic nervous system activity, the ‘rest and digest’ system responsible for regulatory activity at rest, was examined in a study that used existing data to assess adolescent PTSD risk during the COVID-19 lockdown (Linde-Krieger et al., 2024). Participants with higher childhood respiratory sinus arrhythmia (RSA), which reflects both parasympathetic nervous system activity and stress response flexibility, at six years of age showed heightened sensitivity to early adolescent (12 years of age) caregiving quality. Participants with higher RSA and a more aversive caregiving environment (higher caregiver internalising symptoms and lower caregiver attachment), had higher PTS scores measured at age 14–15 years, whilst participants with higher RSA and a more positive caregiving environment had the lowest PTS scores.

Neuroimaging studies indicated structural and functional brain differences, particularly in the amygdala, hippocampus, and prefrontal cortex, which may contribute to PTSD vulnerability, as well as total and domain-specific symptom severity (Heyn & Herringa, 2019; McLaughlin et al., 2014; Ousdal et al., 2020). Several studies examined neuroimaging measures in relation to PTSD. In a study that included children and adolescents (ages 8–18 years) exposed to a range of witnessed and experienced traumas, baseline right orbital gyrus grey matter volume predicted higher and lower one-year PTSD symptom severity in females and males, respectively (Heyn et al., 2022). The presence of a left orbitofrontal cortex lesion and the number of diffuse lesions in children and adolescents (mean age 10.6 years) three months after traumatic brain injury predicted lower one-year hyperarousal but higher avoidance symptoms, respectively (Vasa et al., 2004). Bilateral amygdala activity in response to aversive images, an indication of emotional responding, measured in adolescents (mean age 16.5 years) before the Boston Marathon terrorist attack, was positively associated with attack-related PTS severity (McLaughlin et al., 2014). In a study examining PTSD symptom persistence in youth (8–18 years of age) exposed to a range of traumas, PTSD non-remitters showed decreases in cortical surface area in left ventral prefrontal cortex, supramarginal gyrus and occipital pole, and the right superior parietal and precentral gyri regions of the ventrolateral prefrontal cortex, parietal, and occipital lobes, while remitters exhibited increases in ventromedial prefrontal cortex thickness and left frontal pole surface area over a one-year period (Heyn & Herringa, 2019). Finally, female adolescents (12–16 years of age) who reported less severe childhood trauma exposure and had a stable left alpha asymmetry profile, an electroencephalogram-based measure linked to affect and motivation, were less likely to present with PTSD symptoms or episodes over a two-year follow-up period (Tang et al., 2018).

#### 3.3.2. Psychosocial factors

Studies highlighted the role of psychological factors, such as appraisals, coping strategies, and parental psychopathology, in the development and maintenance of PTSD symptoms (Kolaitis et al., 2011; Marsac et al., 2017; Ostrowski et al., 2006; Schneider & Gudiño, 2018). In children (mean age 10.6 years) hospitalised for traumatic injury, global and trauma-specific threat appraisals assessed within the two weeks following trauma exposure were positively associated with concurrent PTSD symptom severity, but did not predict PTSD symptoms at six or twelve weeks (Marsac et al., 2017). In the same study, children who exhibited PTSD symptoms following injury used more coping strategies to manage their distress. Specifically, avoidance coping was associated with the persistence of symptoms, as it predicted PTSD symptom development over time. In contrast, in children (mean age 11.8 years) who were also admitted to hospital following injury, unhelpful trauma appraisals were positively associated with PTSD symptom severity within four weeks of an injury and six months later (Nixon et al., 2010). In a study of PTSD in children (6–14 years of age) with traumatic brain injury, (Guo et al., 2017) examined whether cognitive factors measured three months post injury were associated with PTSD symptom severity at six months after injury. Sustained, rather than selective attention emerged as a significant predictor of PTSD symptoms whilst greater verbal learning and working memory deficits predicted lesser PTSD symptom severity (Guo et al., 2017). In a study of adolescents (baseline mean age 15.0 years) that collected data at 12, 18 and 24 months after exposure to the Wenchuan earthquake, Geng et al. (2019) found that insomnia was positively associated with PTSD symptoms over time and that the association was mediated by depressive symptoms.

In adolescents (mean age 11.4 years) at risk for violence exposure, greater behavioural inhibition was significantly associated with higher PTSD avoidance symptoms in female participants exposed to violence (Schneider & Gudiño, 2018). The same study found that Latino culture was a positive moderator of the association between behavioural inhibition and PTSD avoidance symptoms i.e. the relationship between behavioural inhibition and PTSD avoidance symptoms strengthened with increasing endorsement of Latino cultural values. In children and adolescents (mean age 13.4 years) hospitalised following injury, maternal PTSD symptoms measured six weeks post their child’s injury were positively associated with their child’s PTSD symptoms at both six weeks and seven months post injury, although the relationship at six weeks became non-significant after excluding dyads where the mother was also a direct victim of the trauma (Ostrowski et al., 2006). In an investigation of both parental psychopathology contributions to PTSD symptoms in children and adolescents (mean age 10.9 years) with MVA exposure, maternal PTSD symptoms at one month follow up predicted child symptoms at three months while paternal and child PTSD symptoms at six months were positively associated (Kolaitis et al., 2011).

## 4. Discussion

**Figure 2.**
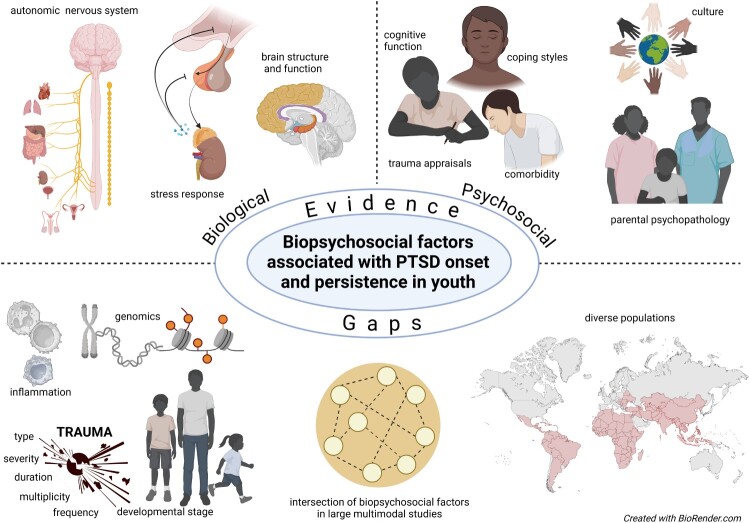
Biopsychosocial factors are implicated in PTSD onset and persistence in children and adolescence. Key biological processes include the stress response, sympathetic and parasympathetic nervous system, and brain structure and function. Psychological comorbidity, cognitive function, trauma appraisals and coping styles were individual-level psychosocial factors. Parental psychopathology and culture also influenced risk. Further research is needed to more closely examine these risk factors and address the gaps in the literature. Studies should be expanded to include mechanisms that have been studied in adults, such as (epi)genetics and inflammation, and should include participants from low- and middle-income countries to more accurately reflect the global trauma burden. The nature, multiplicity, chronicity, frequency and especially timing of trauma exposure should be considered due to the substantial physical, neurobiological, social and psychological development that occurs across youth and adolescence.

The synthesis of results suggests a complex interplay of biopsychosocial factors in the aetiology and course of PTSD in children and adolescents (Figure 2), highlighting the need to consider an integrative approach.

### 4.1. Biological factors

In terms of biological factors, substantial evidence supports the involvement of the stress response in PTS pathophysiology. However, there is a lack of consistency in terms of the direction of these effects, which may be partially explained by study design, participant demographics, trauma type and biological source material. Though the two studies that identified inverse associations between HCC and PTSD diagnosis (Luo et al., 2012)/symptom severity (Straub et al., 2017) differed in the timing of their PTSD assessments (within two months vs. seven months after the trauma), both studies examined participants exposed to acute trauma (earthquake and injury) and found a significant association in only female participants. In contrast, the study that found a positive association was conducted in Syrian refugee children and adolescents exposed to chronic trauma. Though further research is needed, the findings of these high-quality studies suggest that the duration of trauma exposure, as well as sex, may influence HCC findings. These results are also partially in keeping with a meta-analysis of HCC in adult PTSD, which found positive associations with recent or ongoing trauma, but equivocal results for the trajectory of HCC in the aftermath of trauma (Schindler-Gmelch et al., 2024). Urinary cortisol was positively associated with PTSD at six weeks (Delahanty et al., 2005) and seven months (Ostrowski et al., 2007), with the latter finding only significant in male participants. In contrast, a negative association between urinary cortisol and PTS at six months was found but only in participants whose parents also reported high PTS scores (Nugent et al., 2006). Given that all three studies were deemed to be of moderate quality, largely due to uncertainty around the adequacy of sample size and representativeness, there is no difference in the likely reliability of the findings. Furthermore, as urinary cortisol was assessed at hospitalisation following acute trauma in study groups of similar mean ages, no clear inferences about age-associated effects can be drawn. The opposite direction of effects in the four high-quality studies that assessed cortisol in saliva obtained from participants of a reasonably broad range of ages may be due to differences in the type of trauma exposure. The three studies that identified a positive association between cortisol in saliva and PTSD were based on the same parent study sample that examined PTSD due to MVA (Kolaitis et al., 2011; Pervanidou, Kolaitis, Charitaki, Margeli, et al., 2007; Pervanidou, Kolaitis, Charitaki, Lazaropoulou, et al., 2007). The inverse association was identified in participants who had lost a parent in the September 11 terrorist attacks, and may thus also be influenced by potential comorbidity, such as prolonged grief disorder, or the chronic nature of trauma following parental loss (Pfeffer et al., 2007). Ultimately, these results may indicate the importance of dysregulation in the stress response system, rather than the absolute value of stress response markers. Trauma-induced alterations to the setpoint of stress responding may limit the capacity to react in a manner matched to the duration and severity of the stressor, increasing the risk of developing stress-related disorders such as PTSD (Agorastos et al., 2019). Only one study examined this possibility by looking at evoked cortisol responses and found that a diagnosis of PTSD in bereaved children was associated with more effective suppression of cortisol release in response to the administration of the synthetic glucocorticoid dexamethasone (Pfeffer et al., 2007).

Both studies that examined molecular markers of sympathetic nervous system activity identified positive associations between catecholamine concentrations and PTS despite differences in study design and quality. Urinary epinephrine in the acute aftermath of trauma predicted PTSD development whilst plasma noradrenaline in the six months thereafter was associated with PTSD persistence in studies of moderate and high quality, respectively (Delahanty et al., 2005; Pervanidou, Kolaitis, Charitaki, Lazaropoulou, et al., 2007). A meta-analysis of studies examining catecholamine levels in PTSD, which included predominantly adult participants (25 out of 27 studies), did not show significant associations for either plasma noradrenaline or urinary epinephrine but did find that trauma type explained a considerable degree of heterogeneity (Pan et al., 2018). Thus, the consistent direction of effects in the two studies reviewed may be partially due to their both examining PTSD following exposure to traumatic injury. Inconsistent results were obtained in studies focussed on HR, which also reflects sympathetic tone. Though three high-quality studies assessed HR in children of similar ages (mean age 10.6–13.3 years) hospitalised following injury, they yielded no significant associations or otherwise opposite directions of effect on PTS symptoms assessed in the first six weeks following trauma. A meta-analysis of HR and PTSD in adults showed a weak but significant positive association that declined with age and showed a trend towards being stronger with increasing time elapsed between HR measurement and symptom assessment (Morris et al., 2016). It is possible that the more limited timeframe in the studies reviewed, extending up to six months, may have obscured this relationship. The conflicting HR findings could also suggest that, like the cortisol findings, adaptive responding capacity influences PTS risk. Higher childhood RSA, an indication of increased flexibility in stress responding, was associated with a higher risk for PTS symptoms in adolescents with lower-quality caregiving environments (Linde-Krieger et al., 2024). Higher RSA combined with a better caregiving environment was linked to lower PTS scores, supporting a differential susceptibility model that shows heightened sensitivity to environmental influences, both positive and negative. Unlike diathesis-stress models, which focus solely on sensitivity to adversity, this finding suggests new avenues for studying biological factors that promote resilience after trauma.

All the neuroimaging studies reviewed identified significant structural or functional measures that were associated with PTSD risk or persistence, which may reflect the impact of trauma cooccurring with the substantial brain development and maturation that happens across childhood and adolescence (Stevens et al., 2018; Tamnes et al., 2017). The findings included regions, such as the amygdala and prefrontal cortex, in the fear learning and memory network, which have been previously identified as potential contributors to the cognitive and emotion-regulating dysfunctions observed in PTSD studies in both youth and adults (Harnett et al., 2020; Hinojosa et al., 2024). The two studies focussed on functional measures suggest that differences in the neurobiology underpinning emotional responses may be a risk factor for PTSD. McLaughlin et al. identified a positive association between increased amygdala activity in response to aversive imagery and the subsequent development of more severe PTS (McLaughlin et al., 2014). However, the same aversive imagery task was associated with reduced amygdala activity in adult participants with PTSD (Kunimatsu et al., 2020). Given that both studies have relatively small sample sizes and differed in their investigation of amygdala activity as a predictor vs. correlate of PTS, the association between these two variables requires further investigation before inferences can be made. A high-quality electroencephalography study by Tang et al. (2018) found that a stable right alpha asymmetry profile was associated with a higher risk of developing PTSD symptoms (Tang et al., 2018). Though affect and motivation can influence the long-term outcomes of trauma exposure, it is not possible to compare these results to PTSD in adulthood as no studies have examined the trajectory of asymmetry profiles in adult PTSD. In a structural neuroimaging study, PTSD persistence over a one-year study period was associated with a decline in cortical surface areas across five brain regions whilst remission was associated with an increase in ventromedial prefrontal cortex thickness (Heyn & Herringa, 2019). Though causality cannot be inferred from these findings, they suggest that alterations to brain structure may be a correlate of PTS severity in children and adolescents and are interesting for several reasons. The high-quality study did not focus on participants with traumatic brain injury, therefore reducing the potential confounding influences in their analysis. Second, the results suggest that PTSD persistence is associated with an acceleration of the age-related decline in cortical surface area that occurs from late childhood and through adolescence to early adulthood (Tamnes et al., 2017). This aligns with a study in military veterans showing that PTSD predicts a more rapid decline in cortical surface area (Brown et al., 2022). Furthermore, a meta-analysis of studies in children and adolescents linked threat-related traumatic experiences with an acceleration of age-related thinning of the ventromedial prefrontal cortex (Colich et al., 2020). This brain region plays an important role in generation and regulation of negative emotions and is connected to regions such as the hippocampus and amygdala, which have been associated with PTSD in a large-scale consortium study (Logue et al., 2018).

### 4.2. Psychosocial factors

Psychological factors such as appraisals, coping strategies and comorbidity play significant roles in PTSD symptom development and maintenance (Kolaitis et al., 2011; Marsac et al., 2017; Ostrowski et al., 2006; Schneider & Gudiño, 2018). In high-quality studies, unhelpful trauma appraisals increased PTSD symptom severity (Nixon et al., 2010), while avoidance coping predicted symptom persistence (Marsac et al., 2017), highlighting why exposure therapy can be an effective psychotherapeutic tool for PTSD treatment (Yehuda et al., 2015). Insomnia was positively associated with PTSD symptoms and was mediated by depressive symptoms in a high-quality study (Geng et al., 2019), in keeping with population-based study findings that a diagnosis of PTSD substantially increases the odds of comorbidity physical and mental health conditions in adults (Husky et al., 2018). Sustained attention predicted PTSD symptoms, and deficits in verbal learning and working memory were associated with lesser symptom severity (Guo et al., 2017). According to a review of attention deficits in PTSD in adulthood, these findings indicate hyperarousal, a constant state of preparedness for threats, which is hypothesised to be a coping mechanism in PTSD (Punski-Hoogervorst et al., 2023). Despite this, the generalisability of these findings is unclear as the study examined children with traumatic brain injury and was designated as moderate quality due to lack of clarity on sample representativeness and the reasons underlying loss-to-follow-up. Female sex and behavioural inhibition were linked to higher PTSD severity and avoidance symptoms in violence-exposed adolescents, with cultural factors moderating these associations (Schneider & Gudiño, 2018). Though the quality of this study was high, the role of culture in youth PTSD requires further investigation as the more frequent and detailed studies of culture in adult PTSD have indicated that specific cultural phenomena, including stigma, acculturation and discrimination, may more accurately reflect PTSD risk in different population groups (Asnaani & Hall-Clark, 2017). With respect to other social factors, parental psychopathology was significantly associated with their child’s PTSD symptoms in four high-quality studies (Kolaitis et al., 2011; Linde-Krieger et al., 2024; Moser et al., 2023; Nugent et al., 2006). Both paternal and maternal PTSD symptoms were positively associated with child PTSD symptoms, and especially when the mother was directly exposed to the trauma (Kolaitis et al., 2011; Moser et al., 2023; Nugent et al., 2006).

### 4.3. Interactions between biological and psychosocial factors

The biopsychosocial model has become the dominant framework in psychiatry due to its recognition that an individual’s mental health is influenced by their unique biology, psychology and sociocultural context (Lugg (2022). In line with this, all of the 24 studies reviewed collected data on both biological and psychosocial factors (Delahanty et al., 2005; Geng et al., 2019; Guo et al., 2017; Haag et al., 2019; Heyn et al., 2022; Heyn & Herringa, 2019; Kolaitis et al., 2011; Linde-Krieger et al., 2024; Luo et al., 2012; Marsac et al., 2017; McLaughlin et al., 2014; Moser et al., 2023; Nixon et al., 2010; Nugent et al., 2006; Ostrowski et al., 2006; Ostrowski et al., 2007; Pervanidou, Kolaitis, Charitaki, Margeli, et al., 2007; Pervanidou, Kolaitis, Charitaki, Lazaropoulou, et al., 2007; Pfeffer et al., 2007; Schneider & Gudiño, 2018; Smeeth et al., 2023; Straub et al., 2017; Tang et al., 2018; Vasa et al., 2004). However, most studies did not investigate how these factors act together or may be reciprocally related. Instead, the approach of using covariates in regression models or explanations for variation in hierarchical models sought to control for the effects of biological factors when assessing the influence of psychosocial factors and *vice versa*. For example, comorbid symptoms or trauma exposure history were controlled for as psychosocial variables, and age or sex as biological variables. The failure of studies to clarify whether sex as a biological variable or gender as a psychosocial variable was assessed, also makes it challenging to determine the true scope of biological – psychosocial interactions investigated. Hypothesised relationships were not always possible to examine. For example, Marsac et al. (2017) aimed to assess whether trauma appraisals mediated the relationship between HR measured in the acute aftermath of trauma and subsequent PTSD, but the finding that the HR – PTSD symptom association was not significant made examining whether trauma appraisals could explain this relationship redundant (Marsac et al., 2017). Nevertheless, three high-quality studies are particularly noteworthy for their examination of the interactions between biological and psychosocial variables. Nugent et al. (2006) found that lower urinary cortisol and HR at hospitalisation was associated with higher PTS scores in children (mean age 13.19 years) whose parents reported high PTSD scores at six months (Nugent et al., 2006). Linde-Krieger et al. (2024) identified that higher childhood RSA was associated with higher PTSD scores in adolescent participants who reported lower caregiver attachment and experience of more severe caregiver internalising symptoms in early adolescence (Linde-Krieger et al., 2024). The study by Haag et al. (2019), found that the positive association between mean HR when children (6–13 years) recounted a trauma narrative one month post hospitalisation and PTSD symptom scores two months later was only significant when the narrative was jointly recounted with a parent and not when the child was alone. These results are intriguing in so far as they all focus on the role of parents and suggest that parent–child relationships and parent mental health interact with child stress responding and autonomic nervous system function to influence PTSD symptom severity. Despite these promising results, whether biological – psychosocial factor interactions are dependent on developmental stage, the type, timing or severity of trauma, or extend beyond the biopsychosocial factors assessed in these three studies is unclear.

### 4.4. Differences in the scope of biopsychosocial factor studies in youth and adult study groups

There are several notable differences in the scope of variables and populations investigated in studies recruiting youth vs. adults. Regarding the nature of the traumas studied, occupations such as the military or first responders can expose a considerable number of adults to trauma on a chronic basis. In terms of biological factors, genomic and inflammatory marker studies are widely investigated in adult populations (Womersley et al., 2023). Many studies in adult participants have investigated genome-wide (epi)genetic profiles. By pooling multiple cohorts, large research consortia such as the Psychiatric Genetics Consortium PTSD workgroup can access sufficient sample sizes to adequately power complex genome-wide investigations (Nievergelt et al., 2019). In contrast, we did not identify any studies adopting genome-wide approaches that fit our inclusion criteria. We identified only two studies (Pervanidou, Kolaitis, Charitaki, Margeli, et al., 2007; Pervanidou, Kolaitis, Charitaki, Lazaropoulou, et al., 2007), both based on the same study group, that examined inflammatory markers. This is an active area of research in adult PTSD with a recent meta-analysis that pooled data from 8394 adult participants found evidence for higher levels of three immune markers (C-reactive protein, interleukin-6 and tumour necrosis factor alpha) in participants with when compared to participants without PTSD (Peruzzolo et al., 2022). Though this review includes several neuroimaging studies, they do not nearly reach the same sample sizes enabled by the partnership between the Psychiatric Genetics Consortium and the Enhancing NeuroImaging Genetics through Meta-Analysis consortium (Zhu et al., 2023). With respect to psychosocial factors for PTSD in adults, social and partner support is likely to play a larger role than parental support, and comorbidities are more likely to include disorders such as substance or alcohol use disorder (Sareen, 2014).

### 4.5. Limitations and potential biases in the review process

This review has several limitations and potential biases. We excluded unpublished research or research published in a non-commercial form e.g. theses, conference proceedings and pre-prints, which can lead to the omission of valuable findings and perspectives not found in peer-reviewed journals, in turn providing an incomplete or biased representation of the research topic. Focusing solely on longitudinal studies may neglect findings derived from other study designs, like cross-sectional studies, which are more frequently performed and can provide valuable single timepoint insights. This may narrow the review’s perspective and limit the comprehensiveness of its conclusions. Variability in measurement tools used for PTSD diagnosis and severity can affect data comparability and makes the synthesis of findings and the accurate identification of common themes and discrepancies more challenging. Though most studies were of high quality, if findings reported in the lower-quality studies were more heavily influenced by study design or analysis factors, their inclusion could lead to inconsistent results and potential biases, and thus undermine the reliability of the review conclusions. Though there is insufficient focus on resilience and protective factors in childhood PTSD research (Agorastos et al., 2019), this unfortunately fell beyond the scope of what could be adequately covered in the current review. Including studies with a wide range in participant age (including up to 19 years in certain studies) can introduce variability due to developmental differences. This is particularly the case given the sensitive windows during youth when the impact of biological, psychological or social factors may have an exaggerated influence (Stevens et al., 2018). For example, as postnatal development and maturation of the brain is neither necessarily linear nor consistent across all regions, trauma experienced in adolescence may disproportionately impact regions of the brain that undergo a more protracted development, such as the frontal and parietal cortices (Stevens et al., 2018; Tamnes et al., 2017). The influence of psychosocial factors across youth also varies with age. For example, the quality and quantity of parental contact is of critical importance in early childhood but diminishes as the adolescents become increasingly independent (Stevens et al., 2018). Unfortunately, the studies identified in our search generally recruited broader age groups comprising both children and adolescents, which did not allow for age-specific insights. Finally, the scope of our search covered neither the potential impacts of prenatal exposures on the risk of developing PTSD nor the range of protective factors that may allow some individuals to be more resilient to the adverse effects of trauma.

### 4.6. Implications for practice and policy

The review underscores the complex interplay of biological, psychological, and social factors in PTSD, aligning with the biopsychosocial model (Yehuda et al., 2015). The studies reviewed suggest that PTSD in youth is characterised by dynamic neural processes, can show sex-based variations, may be worsened by comorbidities such as cognitive deficits and depression, and is sensitive to social influences like parental psychopathology (Yehuda et al., 2015). This emphasises the need for comprehensive, multidimensional approaches to understanding and addressing PTSD in children and adolescents. Mental health professionals should be trained to recognise and assess biopsychosocial factors for culturally sensitive, contextually relevant, and effective prevention and intervention. Although treatments like cognitive–behavioural therapy and eye movement desensitisation are effective, uncertainties about their optimal use remain (Pfefferbaum et al., 2014). Psychological factors, such as depression and cognitive deficits, should be addressed through evidence-based therapies and the role of social support, including the family environment, and culture should be considered (Yehuda et al., 2015). Insights into biopsychosocial mechanisms, i.e. processes or factors that provide insight into how or why a relationship exists, could inform predictive models, prevention strategies and treatment protocols (Mansour et al., 2023; Yehuda et al., 2015). The review also highlights the lack of global representation in PTSD studies. Only three studies involved LMICs and focussed on specific events (the Wenchuan Earthquake) (Geng et al., 2019; Luo et al., 2012) or populations (Syrian refugee children) (Smeeth et al., 2023). Research on PTSD in LMICs, especially in Sub-Saharan Africa, is limited despite high PTSD rates due to natural disasters, violence, and conflict (Ng et al., 2020). There is a need for research on intervention efficacy and resource adaptation in these regions to address the substantial mental health treatment gap (Sankoh et al., 2018).

### 4.7. Implications for research

This review highlights several gaps in the literature (Figure 2). Future studies should focus on longitudinal, multi-dimensional designs examining the interplay of biological, psychological, and social factors over time, and investigate PTSD and symptom domain mechanisms based on the trauma's nature, timing, chronicity, severity, and multiplicity (Rousseau & Measham, 2007; Yehuda et al., 2015). Establishing universal biological markers is complicated by individual genetic and environmental interactions. However, studies on cell-type-specific molecular pathways, brain genomic profiling, and in vitro modelling are crucial for understanding the genetic and epigenetic factors in PTSD and should include children and adolescents (Iatrou & Daskalakis, 2024). Although it was not the focus of the study, the sex-specific effects of biopsychosocial factors noted in some studies (Guo et al., 2017; Heyn et al., 2022; Ostrowski et al., 2006; Ostrowski et al., 2007; Schneider & Gudiño, 2018; Straub et al., 2017), also require further research. Research should encompass diverse populations to better reflect the global trauma burden, considering the substantial role of contextual and social factors in PTSD. Several studies used existing longitudinal cohort data to examine PTS predictors,(Linde-Krieger et al., 2024; McLaughlin et al., 2014) a powerful approach that should become more feasible as the number of such cohort studies grows.

## 5. Conclusions

Converging evidence points to the significant impact of psychological, neurobiological, social, and cognitive factors, and importantly their interaction, on PTSD symptomatology. Studies have broadly implicated stress response and autonomic nervous system dysregulation, and structural and functional differences in brain regions associated with emotion regulation, learning and memory, and executive function. Findings also emphasised the importance of both screening for and addressing comorbidity, and the role of family and social factors, such as parental PTSD symptoms and socioeconomic status, in the development and severity of PTS. Ultimately, the dynamic nature of PTSD pathophysiology will necessitate comprehensive longitudinal investigations into the intersectionality of biopsychosocial factors, which can then guide individualised treatment plans.

## References

[CIT0001] Agorastos, A., Pervanidou, P., Chrousos, G. P., & Baker, D. G. (2019). Developmental trajectories of early life stress and trauma: A narrative review on neurobiological aspects beyond stress system dysregulation. *Frontiers in Psychiatry*, *10*, 118. 10.3389/fpsyt.2019.00118 30914979 PMC6421311

[CIT0002] Aliev, G., Beeraka, N. M., Nikolenko, V. N., Svistunov, A. A., Rozhnova, T., Kostyuk, S., Cherkesov, I., Gavryushova, L. V., Chekhonatsky, A. A., Mikhaleva, L. M., Somasundaram, S. G., Avila-Rodriguez, M. F., & Kirkland, C. E. (2020). Neurophysiology and psychopathology underlying PTSD and recent insights into the PTSD therapies – a comprehensive review. *Journal of Clinical Medicine*, *9*(9), 2951. 10.3390/jcm9092951 32932645 PMC7565106

[CIT0003] Alisic, E., Zalta, A. K., van Wesel, F., Larsen, S. E., Hafstad, G. S., Hassanpour, K., & Smid, G. E. (2014). Rates of post-traumatic stress disorder in trauma-exposed children and adolescents: Meta-analysis. *British Journal of Psychiatry*, *204*(5), 335–340. 10.1192/bjp.bp.113.131227 24785767

[CIT0004] American Psychiatric Association. (2013). *Diagnostic and statistical manual of mental disorders: DSM-5*. American Psychiatric Association.

[CIT0005] Asnaani, A., & Hall-Clark, B. (2017). Recent developments in understanding ethnocultural and race differences in trauma exposure and PTSD. *Current Opinion in Psychology*, *14*, 96–101. 10.1016/j.copsyc.2016.12.005 28813327

[CIT0006] Brown, E. M., Salat, D. H., Milberg, W. P., Fortier, C. B., & McGlinchey, R. E. (2022). Accelerated longitudinal cortical atrophy in OEF/OIF/OND veterans with severe PTSD and the impact of comorbid TBI. *Human Brain Mapping*, *43*(12), 3694–3705. 10.1002/hbm.25877 35426972 PMC9294300

[CIT0007] Cisler, J. M., & Herringa, R. J. (2021). Posttraumatic stress disorder and the developing adolescent brain. *Biological Psychiatry*, *89*(2), 144–151. 10.1016/j.biopsych.2020.06.001 32709416 PMC7725977

[CIT0008] Cloitre, M., Stolbach, B. C., Herman, J. L., Kolk, B. v. d., Pynoos, R., Wang, J., & Petkova, E. (2009). A developmental approach to complex PTSD: Childhood and adult cumulative trauma as predictors of symptom complexity. *Journal of Traumatic Stress*, *22*(5), 399–408. 10.1002/jts.20444 19795402

[CIT0009] Colich, N. L., Rosen, M. L., Williams, E. S., & McLaughlin, K. A. (2020). Biological aging in childhood and adolescence following experiences of threat and deprivation: A systematic review and meta-analysis. *Psychological Bulletin*, *146*(9), 721–764. 10.1037/bul0000270 32744840 PMC7484378

[CIT0010] Copeland, W. E., Keeler, G., Angold, A., & Costello, E. J. (2007). Traumatic events and posttraumatic stress in childhood. *Archives of General Psychiatry*, *64*(5), 577–584. 10.1001/archpsyc.64.5.577 17485609

[CIT0011] Delahanty, D. L., Nugent, N. R., Christopher, N. C., & Walsh, M. (2005). Initial urinary epinephrine and cortisol levels predict acute PTSD symptoms in child trauma victims. *Psychoneuroendocrinology*, *30*(2), 121–128. 10.1016/j.psyneuen.2004.06.004 15471610

[CIT0012] de Pellegars, A., Cariou, C., Le Floch, M., Duverger, P., Boussicault, G., & Riquin, E. (2024). Risk factors of post-traumatic stress disorder after hospitalization in a pediatric intensive care unit: A systematic literature review. *European Child & Adolescent Psychiatry*, *33*(9), 2991–3001. 10.1007/s00787-023-02141-8 36739584

[CIT0013] Etherington, K. (2007). Working with traumatic stories: From transcriber to witness. *International Journal of Social Research Methodology*, *10*(2), 85–97. 10.1080/13645570701334001

[CIT0014] Geng, F., Liang, Y., Li, Y., Fang, Y., Pham, T. S., Liu, X., & Fan, F. (2019). Bidirectional associations between insomnia, posttraumatic stress disorder, and depressive symptoms among adolescent earthquake survivors: A longitudinal multiwave cohort study. *Sleep*, *42*(11), zsz162. 10.1093/sleep/zsz162 31328781

[CIT0015] Guo, X., Edmed, S. L., Anderson, V., & Kenardy, J. (2017). Neurocognitive predictors of posttraumatic stress disorder symptoms in children 6 months after traumatic brain injury: A prospective study. *Neuropsychology*, *31*(1), 84–92. 10.1037/neu0000305 27617636

[CIT0016] Haag, K., Hiller, R., Peyk, P., Michael, T., Meiser-Stedman, R., Fearon, P., Ehlers, A., & Halligan, S. L. (2019). A longitudinal examination of heart-rate and heart rate variability as risk markers for child posttraumatic stress symptoms in an acute injury sample. *Journal of Abnormal Child Psychology*, *47*(11), 1811–1820. 10.1007/s10802-019-00553-2 31073881 PMC6805807

[CIT0017] Harnett, N. G., Goodman, A. M., & Knight, D. C. (2020). PTSD-related neuroimaging abnormalities in brain function, structure, and biochemistry. *Experimental Neurology*, *330*, 113331. 10.1016/j.expneurol.2020.113331 32343956

[CIT0018] Heyn, S. A., Bailowitz, S., Russell, J. D., & Herringa, R. J. (2022). Sex-based variations of prefrontal structure and longitudinal symptoms in pediatric posttraumatic stress disorder. *Depression and Anxiety*, *39*(12), 902–912. 10.1002/da.23296 36349877 PMC9762118

[CIT0019] Heyn, S. A., & Herringa, R. J. (2019). Longitudinal cortical markers of persistence and remission of pediatric PTSD. *NeuroImage: Clinical*, *24*, 102028. 10.1016/j.nicl.2019.102028 31670153 PMC6831901

[CIT0020] Hillis, S., Mercy, J., Amobi, A., & Kress, H. (2016). Global prevalence of past-year violence against children: A systematic review and minimum estimates. *Pediatrics*, *137*(3), e20154079. 10.1542/peds.2015-4079 26810785 PMC6496958

[CIT0021] Hinojosa, C. A., George, G. C., & Ben-Zion, Z. (2024). Neuroimaging of posttraumatic stress disorder in adults and youth: Progress over the last decade on three leading questions of the field. *Molecular Psychiatry*, *29*(10), 3223–3244. 10.1038/s41380-024-02558-w 38632413 PMC11449801

[CIT0022] Hu, Y., Chu, X., Urosevich, T. G., Hoffman, S. N., Kirchner, H. L., Adams, R. E., Dugan, R. J., Boscarino, J. J., Shi, W., Withey, C. A., Figley, C. R., & Boscarino, J. A. (2020). Predictors of current DSM-5 PTSD diagnosis and symptom severity Among deployed veterans: Significance of predisposition, stress exposure, and genetics. *Neuropsychiatric Disease and Treatment*, *16*, 43–54. 10.2147/NDT.S228802 32021198 PMC6956712

[CIT0023] Husky, M. M., Mazure, C. M., & Kovess-Masfety, V. (2018). Gender differences in psychiatric and medical comorbidity with post-traumatic stress disorder. *Comprehensive Psychiatry*, *84*, 75–81. 10.1016/j.comppsych.2018.04.007 29723769

[CIT0024] Iatrou, A., & Daskalakis, N. P. (2024). Unraveling the cell-type-specific molecular pathways of PTSD: Integrating GWAS with brain genomic profiling and in vitro modeling. *Neuropsychopharmacology*, *49*(1), 303–304. 10.1038/s41386-023-01698-x 37580460 PMC10700486

[CIT0025] Knipscheer, J., Sleijpen, M., Frank, L., de Graaf, R., Kleber, R., ten Have, M., & Dückers, M. (2020). Prevalence of potentially traumatic events, other life events and subsequent reactions indicative for posttraumatic stress disorder in The Netherlands: A general population study based on the trauma screening questionnaire. *International Journal of Environmental Research and Public Health*, *17*(5), 1725. 10.3390/ijerph17051725 32155752 PMC7084195

[CIT0026] Koenen, K. C., Ratanatharathorn, A., Ng, L., McLaughlin, K. A., Bromet, E. J., Stein, D. J., Karam, E. G., Meron Ruscio, A., Benjet, C., Scott, K., Atwoli, L., Petukhova, M., Lim, C. C. W., Aguilar-Gaxiola, S., Al-Hamzawi, A., Alonso, J., Bunting, B., Ciutan, M., de Girolamo, G., … Kessler, R. C. (2017). Posttraumatic stress disorder in the world mental health surveys. *Psychological Medicine*, *47*(13), 2260–2274. 10.1017/S0033291717000708 28385165 PMC6034513

[CIT0027] Kolaitis, G., Giannakopoulos, G., Liakopoulou, M., Pervanidou, P., Charitaki, S., Mihas, C., Ferentinos, S., Papassotiriou, I., Chrousos, G. P., & Tsiantis, J. (2011). Predicting pediatric posttraumatic stress disorder after road traffic accidents: The role of parental psychopathology. *Journal of Traumatic Stress*, *24*(4), 414–421. 10.1002/jts.20667 21812037

[CIT0028] Kunimatsu, A., Yasaka, K., Akai, H., Kunimatsu, N., & Abe, O. (2020). MRI findings in posttraumatic stress disorder. *Journal of Magnetic Resonance Imaging*, *52*(2), 380–396. 10.1002/jmri.26929 31515885

[CIT0029] Lewis, S. J., Arseneault, L., Caspi, A., Fisher, H. L., Matthews, T., Moffitt, T. E., Odgers, C. L., Stahl, D., Teng, J. Y., & Danese, A. (2019). The epidemiology of trauma and post-traumatic stress disorder in a representative cohort of young people in England and Wales. *The Lancet Psychiatry*, *6*(3), 247–256. 10.1016/S2215-0366(19)30031-8 30798897 PMC6384243

[CIT0030] Linde-Krieger, L. B., Rudd, K. L., Aringer, A. S., & Yates, T. M. (2024). A longitudinal investigation of caregiving and adolescent post-traumatic stress symptoms during COVID-19: Evidence for high resting RSA as a susceptibility factor. *Psychological Medicine*, *54*(10), 2457–2467. 10.1017/S003329172400059X 38481341 PMC13399547

[CIT0031] Logue, M. W., van Rooij, S. J. H., Dennis, E. L., Davis, S. L., Hayes, J. P., Stevens, J. S., Densmore, M., Haswell, C. C., Ipser, J., Koch, S. B. J., Korgaonkar, M., Lebois, L. A. M., Peverill, M., Baker, J. T., Boedhoe, P. S. W., Frijling, J. L., Gruber, S. A., Harpaz-Rotem, I., Jahanshad, N., … Morey, R. A. (2018). Smaller hippocampal volume in posttraumatic stress disorder: A multisite ENIGMA-PGC study: Subcortical volumetry results from posttraumatic stress disorder consortia. *Biological Psychiatry*, *83*(3), 244–253. 10.1016/j.biopsych.2017.09.006 29217296 PMC5951719

[CIT0032] Lugg, W. (2022). The biopsychosocial model – History, controversy and engel. *Australasian Psychiatry*, *30*(1), 55–59. 10.1177/10398562211037333 34748708

[CIT0033] Luo, H., Hu, X., Liu, X., Ma, X., Guo, W., Qiu, C., Wang, Y., Wang, Q., Zhang, X., Zhang, W., Hannum, G., Zhang, K., Liu, X., & Li, T. (2012). Hair cortisol level as a biomarker for altered hypothalamic-pituitary-adrenal activity in female adolescents with posttraumatic stress disorder after the 2008 Wenchuan earthquake. *Biological Psychiatry*, *72*(1), 65–69. 10.1016/j.biopsych.2011.12.020 22305287

[CIT0034] Magwai, T., & Xulu, K. R. (2022). Physiological genomics plays a crucial role in response to stressful life events, the development of aggressive behaviours, and post-traumatic stress disorder (PTSD). *Genes*, *13*(2), 300. 10.3390/genes13020300 35205345 PMC8871735

[CIT0035] Mansour, M., Joseph, G. R., Joy, G. K., Khanal, S., Dasireddy, R. R., Menon, A., Barrie Mason, I., Kataria, J., Patel, T., & Modi, S. (2023). Post-traumatic stress disorder: A narrative review of pharmacological and psychotherapeutic interventions. *Cureus*. *15*(9), e44905. 10.7759/cureus.44905 37814755 PMC10560516

[CIT0036] Marsac, M. L., Kassam-Adams, N., Delahanty, D. L., Ciesla, J., Weiss, D., Widaman, K. F., & Barakat, L. P. (2017). An initial application of a biopsychosocial framework to predict posttraumatic stress following pediatric injury. *Health Psychology*, *36*(8), 787–796. 10.1037/hea0000508 28650199 PMC5673123

[CIT0037] McClendon, J., Perkins, D., Copeland, L. A., Finley, E. P., & Vogt, D. (2019). Patterns and correlates of racial/ethnic disparities in posttraumatic stress disorder screening among recently separated veterans. *Journal of Anxiety Disorders*, *68*, 102145. 10.1016/j.janxdis.2019.102145 31550626

[CIT0038] McLaughlin, K. A., Busso, D. S., Duys, A., Green, J. G., Alves, S., Way, M., & Sheridan, M. A. (2014). Amygdala response to negative stimuli predicts PTSD symptom onset following a terrorist attack: Research article: Amygdala reactivity and PTSD onset. *Depression and Anxiety*, *31*(10), 834–842. 10.1002/da.22284 24995938 PMC4205168

[CIT0039] Memarzia, J., Walker, J., & Meiser-Stedman, R. (2021). Psychological peritraumatic risk factors for post-traumatic stress disorder in children and adolescents: A meta-analytic review. *Journal of Affective Disorders*, *282*, 1036–1047. 10.1016/j.jad.2021.01.016 33601676

[CIT0040] Misganaw, B., Guffanti, G., Lori, A., Abu-Amara, D., Flory, J. D., Hammamieh, R., Gautam, A., Yang, R., Daigle, B. J., Hood, L., Wang, K., Lee, I., Mellon, S. H., Wolkowitz, O. M., Mueller, S., Yehuda, R., Jett, M., Marmar, C. R., Ressler, K. J., & Doyle, F. J. (2019). Polygenic risk associated with post-traumatic stress disorder onset and severity. *Translational Psychiatry*, *9*(1), 165. 10.1038/s41398-019-0497-3 31175274 PMC6555815

[CIT0041] Mitchell, R., Brennan, K., Curran, D., Hanna, D., & Dyer, K. F. W. (2017). A meta-analysis of the association between appraisals of trauma and posttraumatic stress in children and adolescents. *Journal of Traumatic Stress*, *30*(1), 88–93. 10.1002/jts.22157 28103414

[CIT0042] Morris, A., Gabert-Quillen, C., & Delahanty, D. (2012). The association between parent PTSD/depression symptoms and child PTSD symptoms: A meta-analysis. *Journal of Pediatric Psychology*, *37*(10), 1076–1088. 10.1093/jpepsy/jss091 23019132

[CIT0043] Morris, M. C., Hellman, N., Abelson, J. L., & Rao, U. (2016). Cortisol, heart rate, and blood pressure as early markers of PTSD risk: A systematic review and meta-analysis. *Clinical Psychology Review*, *49*, 79–91. 10.1016/j.cpr.2016.09.001 27623149 PMC5079809

[CIT0044] Moser, D. A., Graf, S., Glaus, J., Urben, S., Jouabli, S., Pointet Perrizolo, V., Suardi, F., Robinson, J., Rusconi Serpa, S., Plessen, K. J., & Schechter, D. S. (2023). On the complex and dimensional relationship of maternal posttraumatic stress disorder during early childhood and child outcomes at school-age. *European Psychiatry*, *66*(1), e20. 10.1192/j.eurpsy.2023.8 36734250 PMC9970153

[CIT0045] Newnham, E. A., Mergelsberg, E. L. P., Chen, Y., Kim, Y., Gibbs, L., Dzidic, P. L., Ishida DaSilva, M., Chan, E. Y. Y., Shimomura, K., Narita, Z., Huang, Z., & Leaning, J. (2022). Long term mental health trajectories after disasters and pandemics: A multilingual systematic review of prevalence, risk and protective factors. *Clinical Psychology Review*, *97*, 102203. 10.1016/j.cpr.2022.102203 36162175

[CIT0046] Ng, L. C., Stevenson, A., Kalapurakkel, S. S., Hanlon, C., Seedat, S., Harerimana, B., Chiliza, B., & Koenen, K. C. (2020). National and regional prevalence of posttraumatic stress disorder in sub-Saharan Africa: A systematic review and meta-analysis. *PLOS Medicine*, *17*(5), e1003090. 10.1371/journal.pmed.1003090 32413027 PMC7228043

[CIT0047] Nievergelt, C. M., Maihofer, A. X., Klengel, T., Atkinson, E. G., Chen, C.-Y., Choi, K. W., Coleman, J. R. I., Dalvie, S., Duncan, L. E., Gelernter, J., Levey, D. F., Logue, M. W., Polimanti, R., Provost, A. C., Ratanatharathorn, A., Stein, M. B., Torres, K., Aiello, A. E., Almli, L. M., … Koenen, K. C. (2019). International meta-analysis of PTSD genome-wide association studies identifies sex- and ancestry-specific genetic risk loci. *Nature Communications*, *10*(1), 1–16. 10.1038/s41467-019-12576-w PMC6783435 31594949

[CIT0048] Nixon, R. D. V., Nehmy, T. J., Ellis, A. A., Ball, S.-A., Menne, A., & McKinnon, A. C. (2010). Predictors of posttraumatic stress in children following injury: The influence of appraisals, heart rate, and morphine use. *Behaviour Research and Therapy*, *48*(8), 810–815. 10.1016/j.brat.2010.05.002 20537316

[CIT0049] Nugent, N. R., Ostrowski, S., Christopher, N. C., & Delahanty, D. L. (2006). Parental posttraumatic stress symptoms as a moderator of child’s acute biological response and subsequent posttraumatic stress symptoms in pediatric injury patients. *Journal of Pediatric Psychology*, *32*(3), 309–318. 10.1093/jpepsy/jsl005 16762993

[CIT0050] O’Donnell, M. L., Alkemade, N., Nickerson, A., Creamer, M., McFarlane, A. C., Silove, D., Bryant, R. A., & Forbes, D. (2014). Impact of the diagnostic changes to post-traumatic stress disorder for DSM-5 and the proposed changes to ICD-11. *British Journal of Psychiatry*, *205*(3), 230–235. 10.1192/bjp.bp.113.135285 24809400

[CIT0051] Ostrowski, S. A., Christopher, N. C., & Delahanty, D. L. (2006). Brief report: The impact of maternal posttraumatic stress disorder symptoms and child gender on risk for persistent posttraumatic stress disorder symptoms in child trauma victims. *Journal of Pediatric Psychology*, *32*(3), 338–342. 10.1093/jpepsy/jsl003 16717137

[CIT0052] Ostrowski, S. A., Christopher, N. C., Van Dulmen, M. H. M., & Delahanty, D. L. (2007). Acute child and mother psychophysiological responses and subsequent PTSD symptoms following a child’s traumatic event. *Journal of Traumatic Stress*, *20*(5), 677–687. 10.1002/jts.20286 17955521

[CIT0053] Ousdal, O. T., Milde, A. M., Hafstad, G. S., Hodneland, E., Dyb, G., Craven, A. R., Melinder, A., Endestad, T., & Hugdahl, K. (2020). The association of PTSD symptom severity with amygdala nuclei volumes in traumatized youths. *Translational Psychiatry*, *10*(1), 288. 10.1038/s41398-020-00974-4 32807799 PMC7431855

[CIT0054] Ouzzani, M., Hammady, H., Fedorowicz, Z., & Elmagarmid, A. (2016). Rayyan – a web and mobile app for systematic reviews. *Systematic Reviews*, *5*(1), 210. 10.1186/s13643-016-0384-4 27919275 PMC5139140

[CIT0055] Page, M. J., McKenzie, J. E., Bossuyt, P. M., Boutron, I., Hoffmann, T. C., Mulrow, C. D., Shamseer, L., Tetzlaff, J. M., Akl, E. A., Brennan, S. E., Chou, R., Glanville, J., Grimshaw, J. M., Hróbjartsson, A., Lalu, M. M., Li, T., Loder, E. W., Mayo-Wilson, E., McDonald, S., … Moher, D. (2021). The PRISMA 2020 statement: An updated guideline for reporting systematic reviews. *BMJ*, 372, n71. 10.1136/bmj.n71 33782057 PMC8005924

[CIT0056] Pan, X., Kaminga, A. C., Wen, S. W., & Liu, A. (2018). Catecholamines in post-traumatic stress disorder: A systematic review and meta-analysis. *Frontiers in Molecular Neuroscience*, *11*, 450. 30564100 10.3389/fnmol.2018.00450 PMC6288600

[CIT0057] Peruzzolo, T. L., Pinto, J. V., Roza, T. H., Shintani, A. O., Anzolin, A. P., Gnielka, V., Kohmann, A. M., Marin, A. S., Lorenzon, V. R., Brunoni, A. R., Kapczinski, F., & Passos, I. C. (2022). Inflammatory and oxidative stress markers in post-traumatic stress disorder: A systematic review and meta-analysis. *Molecular Psychiatry*, *27*(8), 3150–3163. 10.1038/s41380-022-01564-0 35477973

[CIT0058] Pervanidou, P., Kolaitis, G., Charitaki, S., Lazaropoulou, C., Papassotiriou, I., Hindmarsh, P., Bakoula, C., Tsiantis, J., & Chrousos, G. P. (2007). The natural history of neuroendocrine changes in pediatric posttraumatic stress disorder (PTSD) after motor vehicle accidents: Progressive divergence of noradrenaline and cortisol concentrations over time. *Biological Psychiatry*, *62*(10), 1095–1102. 10.1016/j.biopsych.2007.02.008 17624319

[CIT0059] Pervanidou, P., Kolaitis, G., Charitaki, S., Margeli, A., Ferentinos, S., Bakoula, C., Lazaropoulou, C., Papassotiriou, I., Tsiantis, J., & Chrousos, G. P. (2007). Elevated morning serum interleukin (IL)-6 or evening salivary cortisol concentrations predict posttraumatic stress disorder in children and adolescents six months after a motor vehicle accident. *Psychoneuroendocrinology*, *32*(8–10), 991–999. 10.1016/j.psyneuen.2007.07.001 17825995

[CIT0060] Pfeffer, C. R., Altemus, M., Heo, M., & Jiang, H. (2007). Salivary cortisol and psychopathology in children bereaved by the September 11, 2001 terror attacks. *Biological Psychiatry*, *61*(8), 957–965. 10.1016/j.biopsych.2006.07.037 17137565

[CIT0061] Pfefferbaum, B., Newman, E., & Nelson, S. D. (2014). Mental health interventions for children exposed to disasters and terrorism. *Journal of Child and Adolescent Psychopharmacology*, *24*(1), 24–31. 10.1089/cap.2013.0061 24494722

[CIT0062] Punski-Hoogervorst, J. L., Engel-Yeger, B., & Avital, A. (2023). Attention deficits as a key player in the symptomatology of posttraumatic stress disorder: A review. *Journal of Neuroscience Research*, *101*(7), 1068–1085. 10.1002/jnr.25177 36807926

[CIT0063] Ross, L. E., Grigoriadis, S., Mamisashvili, L., Koren, G., Steiner, M., Dennis, C-L, Cheung, A., & Mousmanis, P. (2011). Quality assessment of observational studies in psychiatry: An example from perinatal psychiatric research. *International Journal of Methods in Psychiatric Research*, *20*(4), 224–234. 10.1002/mpr.356 22113965 PMC6878491

[CIT0064] Rousseau, C., & Measham, T. (2007). Posttraumatic suffering as a source of transformation: A clinical perspective. In L. J. Kirmayer, R. Lemelson, & M. Barad (Eds.), *Understanding trauma* (pp. 275–294). Cambridge University Press. 10.1017/CBO9780511500008.019.

[CIT0065] Sankoh, O., Sevalie, S., & Weston, M. (2018). Mental health in Africa. *The Lancet Global Health*, *6*(9), e954–e955. 10.1016/S2214-109X(18)30303-6 30103990

[CIT0066] Sareen, J. (2014). Posttraumatic stress disorder in adults: Impact, comorbidity, risk factors, and treatment. *The Canadian Journal of Psychiatry*, *59*(9), 460–467. 10.1177/070674371405900902 25565692 PMC4168808

[CIT0067] Schindler-Gmelch, L., Capito, K., Steudte-Schmiedgen, S., Kirschbaum, C., & Berking, M. (2024). Hair cortisol research in posttraumatic stress disorder – 10 years of insights and open questions. A systematic review. *Current Neuropharmacology*, *22*(10), 1697–1719. 10.2174/1570159X21666230807112425 37550910 PMC11284720

[CIT0068] Schneider, A., & Gudiño, O. G. (2018). Predicting avoidance symptoms in U.S. Latino youth exposed to community violence: The role of cultural values and behavioral inhibition. *Journal of Traumatic Stress*, *31*(4), 509–517. 10.1002/jts.22313 30058738

[CIT0069] Smeeth, D., McEwen, F. S., Popham, C. M., Karam, E. G., Fayyad, J., Saab, D., Rieder, M. J., Elzagallaai, A. A., van Uum, S., & Pluess, M. (2023). War exposure, post-traumatic stress symptoms and hair cortisol concentrations in Syrian refugee children. *Molecular Psychiatry*, *28*(2), 647–656. 10.1038/s41380-022-01859-2 36385169 PMC9908541

[CIT0070] Stevens, J. S., van Rooij, S. J. H., & Jovanovic, T. (2018). Developmental contributors to trauma response: The importance of sensitive periods, early environment, and sex differences. *Current Topics in Behavioral Neurosciences*, *38*, 1–22. 27830573 10.1007/7854_2016_38 PMC5425320

[CIT0071] Straub, J., Klaubert, L. M., Schmiedgen, S., Kirschbaum, C., & Goldbeck, L. (2017). Hair cortisol in relation to acute and post-traumatic stress symptoms in children and adolescents. *Anxiety, Stress, & Coping*, *30*(6), 661–670. 10.1080/10615806.2017.1355458 28745078

[CIT0072] Tamnes, C. K., Herting, M. M., Goddings, A.-L., Meuwese, R., Blakemore, S.-J., Dahl, R. E., Güroğlu, B., Raznahan, A., Sowell, E. R., Crone, E. A., & Mills, K. L. (2017). Development of the cerebral cortex across adolescence: A multisample study of inter-related longitudinal changes in cortical volume, surface area, and thickness. *The Journal of Neuroscience*, *37*(12), 3402–3412. 10.1523/JNEUROSCI.3302-16.2017 28242797 PMC5373125

[CIT0073] Tang, A., Miskovic, V., Lahat, A., Tanaka, M., MacMillan, H., Van Lieshout, R. J., & Schmidt, L. A. (2018). Trajectories of resting frontal brain activity and psychopathology in female adolescents exposed to child maltreatment. *Developmental Psychobiology*, *60*(1), 67–77. 10.1002/dev.21585 29130493

[CIT0074] Triantafyllou, C., & Matziou, V. (2019). Aggravating factors and assessment tools for posttraumatic stress disorder in children after hospitalization. *Psychiatriki*, *30*(3), 256–266. 10.22365/jpsych.2019.303.256 31685457

[CIT0075] Vasa, R. A., Grados, M., Slomine, B., Herskovits, E. H., Thompson, R. E., Salorio, C., Christensen, J., Wursta, C., Riddle, M. A., & Gerring, J. P. (2004). Neuroimaging correlates of anxiety after pediatric traumatic brain injury. *Biological Psychiatry*, *55*(3), 208–216. 10.1016/S0006-3223(03)00708-X 14744460

[CIT0076] Williamson, V., Creswell, C., Fearon, P., Hiller, R. M., Walker, J., & Halligan, S. L. (2017). The role of parenting behaviors in childhood post-traumatic stress disorder: A meta-analytic review. *Clinical Psychology Review*, *53*, 1–13. 10.1016/j.cpr.2017.01.005 28137661

[CIT0077] Womersley, J. S., du Plessis, M., Greene, M. C., van den Heuwel, L. L., Kinyanda, E., & Seedat, S. (2023). Advances in the molecular neurobiology of posttraumatic stress disorder from global contexts: A systematic review of longitudinal studies. *Cambridge Prisms: Global Mental Health*, *10*, e62. 10.1017/gmh.2023.53 37854422 PMC10579657

[CIT0078] Woolgar, F., Garfield, H., Dalgleish, T., & Meiser-Stedman, R. (2022). Systematic review and meta-analysis: Prevalence of posttraumatic stress disorder in trauma-exposed preschool-aged children. *Journal of the American Academy of Child & Adolescent Psychiatry*, *61*(3), 366–377. 10.1016/j.jaac.2021.05.026 34242737 PMC8885427

[CIT0079] Yehuda, R., Hoge, C. W., McFarlane, A. C., Vermetten, E., Lanius, R. A., Nievergelt, C. M., Hobfoll, S. E., Koenen, K. C., Neylan, T. C., & Hyman, S. E. (2015). Post-traumatic stress disorder. *Nature Reviews Disease Primers*, *1*(1), 15057. 10.1038/nrdp.2015.57 27189040

[CIT0080] Yu, H., Nie, C., Zhou, Y., Wang, X., Wang, H., & Shi, X. (2020). Epidemiological characteristics and risk factors of posttraumatic stress disorder in Chinese children after exposure to an injury. *Disaster Medicine and Public Health Preparedness*, *14*(4), 486–493. 10.1017/dmp.2019.93 31610821

[CIT0081] Zhu, X., Kim, Y., Ravid, O., He, X., Suarez-Jimenez, B., Zilcha-Mano, S., Lazarov, A., Lee, S., Abdallah, C. G., Angstadt, M., Averill, C. L., Baird, C. L., Baugh, L. A., Blackford, J. U., Bomyea, J., Bruce, S. E., Bryant, R. A., Cao, Z., Choi, K., … Morey, R. A. (2023). Neuroimaging-based classification of PTSD using data-driven computational approaches: A multisite big data study from the ENIGMA-PGC PTSD consortium. *Neuroimage*, *283*, 120412. 10.1016/j.neuroimage.2023.120412 37858907 PMC10842116

